# Microplasma-assisted hydrogel fabrication: A novel method for gelatin-graphene oxide nano composite hydrogel synthesis for biomedical application

**DOI:** 10.7717/peerj.3498

**Published:** 2017-06-27

**Authors:** Mantosh Kumar Satapathy, Wei-Hung Chiang, Er-Yuan Chuang, Chih-Hwa Chen, Jia-Liang Liao, Huin-Ning Huang

**Affiliations:** 1 Graduate Institute of Biomedical Materials and Tissue Engineering, Taipei Medical University, Taipei, Taiwan; 2 Department of Chemical Engineering, National Taiwan University of Science and Technology, Taipei, Taiwan; 3 Bone and Joint Research Center, Department of Orthopedics, Taipei Medical University Hospital, School of Medicine, College of Medicine, Taipei, Taiwan; 4 School of Biomedical Engineering, College of Biomedical Engineering, Taipei Medical University, Taipei, Taiwan

**Keywords:** Argon microplasma, Gelatin, Graphene oxide, Cross-linking, Hydrogel, Biocompatibility, Tissue engineering

## Abstract

Toxicity issues and biocompatibility concerns with traditional classical chemical cross-linking processes prevent them from being universal approaches for hydrogel fabrication for tissue engineering. Physical cross-linking methods are non-toxic and widely used to obtain cross-linked polymers in a tunable manner. Therefore, in the current study, argon micro-plasma was introduced as a neutral energy source for cross-linking in fabrication of the desired gelatin-graphene oxide (gel-GO) nanocomposite hydrogel scaffolds. Argon microplasma was used to treat purified gelatin (8% w/v) containing 0.1∼1 wt% of high-functionality nano-graphene oxide (GO). Optimized plasma conditions (2,500 V and 8.7 mA) for 15 min with a gas flow rate of 100 standard cm^3^/min was found to be most suitable for producing the gel-GO nanocomposite hydrogels. The developed hydrogel was characterized by the degree of cross-linking, FTIR spectroscopy, SEM, confocal microscopy, swelling behavior, contact angle measurement, and rheology. The cell viability was examined by an MTT assay and a live/dead assay. The pore size of the hydrogel was found to be 287 ± 27 µm with a contact angle of 78° ± 3.7°. Rheological data revealed improved storage as well as a loss modulus of up to 50% with tunable viscoelasticity, gel strength, and mechanical properties at 37 °C temperature in the microplasma-treated groups. The swelling behavior demonstrated a better water-holding capacity of the gel-GO hydrogels for cell growth and proliferation. Results of the MTT assay, microscopy, and live/dead assay exhibited better cell viability at 1% (w/w) of high-functionality GO in gelatin. The highlight of the present study is the first successful attempt of microplasma-assisted gelatin-GO nano composite hydrogel fabrication that offers great promise and optimism for further biomedical tissue engineering applications.

## Introduction

Tissue engineering is an emerging field that exists at the interface of material science, chemical engineering, and life science to develop alternatives to restore, improve and maintain diseased or damaged tissues (Lanza, Langer & Vacanti, 2011); thus, it represents a fascinating trend in regenerative medicine. Broadly, the therapeutic approach in orthopedic tissue engineering focuses on the regeneration of a variety of connective tissues such as bone, cartilage, ligament, tendons, and muscle tissues (Lu & Thomopoulos, 2013). Amongst the main challenge in orthopedic tissue engineering are the selection of appropriate cells (differentiated or progenitor cells) followed by fabrication and utilization of biocompatible and mechanically suitable scaffolds with enhanced potential to target major unresolved issues from the past (Kuo et al., 2006).

Despite the intrinsic capability of connective tissues in the body to regenerate, they fail to regenerate themselves during injury or some diseases that ultimately lead to the loss of, or damage to, connective tissues. Connective tissue degeneration is one of the most common causes of pain, limited movement, deformity, and eventually progressive disability if not treated in time. Traditional surgical reconstruction fails to fully repair lost connective tissues and often causes donor site morbidity (Cezar & Mooney, 2015).

Recently, polymer-based scaffold fabrication gained popularity in tissue engineering during scaffold designing (Piskin, 1995; Ji et al., 2006) for repairing and regenerating desired tissues. Polymeric hydrogels, due to their unique biocompatibility and desirable physical characteristics, have a long history of use as scaffold material of choice for tissue engineering. Besides serving as matrices for tissue engineering and regenerative medicine, these polymer based hydrogels are capable of mimicking the extracellular matrix topography and can thus facilitate the delivery of required bioactive agents that promote tissue regeneration (Amini, Laurencin & Nukavarapu, 2012; Park, 2011). Gelatin is a suitable polymer that has been extensively used in tissue engineering hydrogel scaffolds fabrication due to its high viscosity, density, excellent biocompatibility and tunable properties (Golden & Tien, 2007). More interestingly, gelatin-based materials due to the presence of arginine-glycine-aspartic acid (RGD) adhesion peptide sequences are promising scaffolds for cell-based repair and facilitate cellular attachment, proliferation, and growth (Shin, Jo & Mikos, 2003) with better biocompatibility (Elzoghby, 2013; Hersel, Dahmen & Kessler, 2003; Ladage et al., 2011); and have already been approved by the US Food and Drug Administration (US-FDA) for clinical use. The polymerization of gelatin occurs at mild conditions (room temperature, neutral pH, in aqueous environments) which facilitate cross-linking and hydrogel formation (Aubin et al., 2010; Nichol et al., 2010). However, limitations of gelatin, such as poor mechanical strength and easy to get contaminate, are needed to be addressed to make it an ideal scaffold material for tissue engineering.

Recently, graphene oxide (GO) has been gaining popularity as additive material along with biopolymers to improve their biocompatibility, mechanical strength, cell adhesion, and proliferation properties, specifically for various tissue engineering applications (Chang et al., 2011). When mixed with gelatin, due to presence of oxygen-containing hydrophilic groups in GO aromatic chains reduces the irreversible agglomeration of graphite sheets through π–π stacking and Van der Waals interactions for ease of making homogenous dispersions with gelatin solutions (Ge et al., 2012; Liu et al., 2011).

The effective fabrication of gelatin-GO based hydrogels is still lacking due to less well known mechanisms of cross-linking behind it. To date, chemical cross-linking by methylacrylate, glutaraldehyde, carbodiimide etc. are established chemical cross-linking techniques (Barbetta et al., 2006; Farris, Song & Huang, 2009; Park et al., 2002). However, physical cross-linking methods may provide easier and non-toxic way over chemical methods to obtain tunable cross-linked polymers (Rowan et al., 2002). Plasma-induced (Gomathi, Sureshkumar & Neogi, 2008) (highly energetic fourth state of matter) cross-linking could be a better choice than chemical cross-linking methods due to its nontoxic chemical free nature. Also, the shorter duration of cross-linking process by plasma technique (Kitching, Pan & Ratner, 2004) could make it novel and unique process for scale-up during commercial industrial applications and could also provide sterile products with better flexibility (Ohl & Schröder, 1999) for clinical applications than those of non-plasma techniques. Furthermore, no study has been reported using plasma for the cross linking and fabrication of Gelatin-GO based nano composite hydrogel systems.

Lesser dimensions microplasmas have been reported as useful tools for materials synthesis and processing previously. Confining the plasma to a micronscale leads to its stability at atmospheric pressure (Mariotti & Sankaran, 2010), making microplasma easy to implement and highly desirable tool for industrial applications. The generated gas discharges contain a high density of energetic electrons (>10 eV) that allows efficient material synthesis and processing. The previous reports also have demonstrated the feasibility of the microplasma-based process to produce metal (Chiang & Sankaran, 2009; Chiang & Sankaran, 2008) and semiconductor nanoparticles (Sankaran et al., 2005), oxides (Mariotti, Bose & Ostrikov, 2009), and carbon nanostructures (Ghezzi et al., 2014; Luo et al., 2016).

To the present, in the biomedical field, plasma research has been used for various applications such as surface sterilization (Kvam et al., 2012), promotion of hemostasis (Schmidt et al., 2015), enhancement of tissue regeneration (Lee et al., 2015), acceleration of wound healing (Arjunan & Clyne, 2011), and anticancer therapy (Fridman et al., 2008; Schlegel, Köritzer & Boxhammer, 2013). However, more research is needed to establish plasma induced hydrogels synthesis and its successful implementation in biomedical tissue engineering (with or without cell based therapy) scaffold fabrication for tissue regeneration and repair.

**Figure 1:**
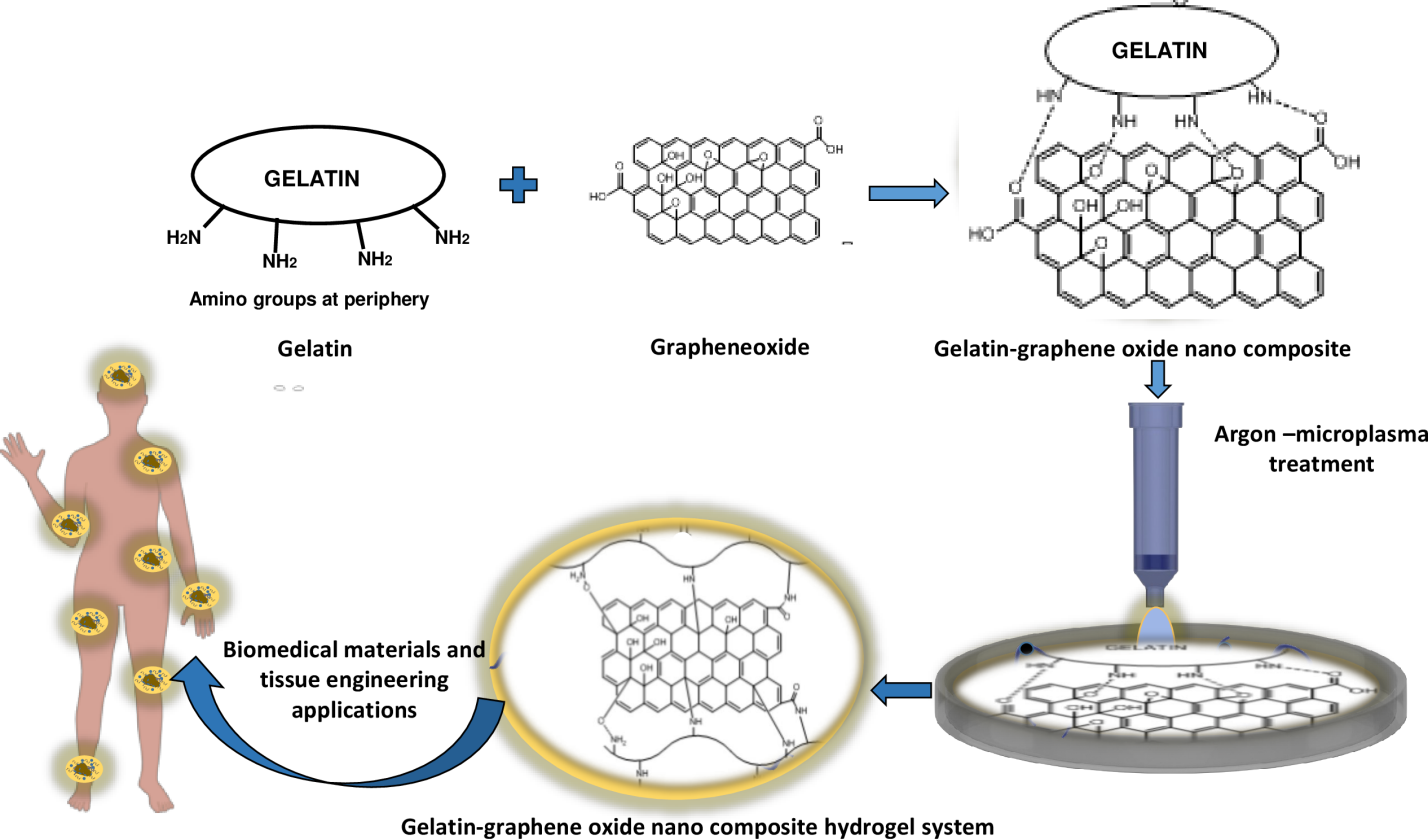
Schematic presentation of Ar- microplasma mediated gel-GO nano composite hydrogel synthesis and its’ biomedical applications (PRISMA flow diagram). Ar- microplasma helps in formation of gel-GO hydrogel system by free radical initiated molecular interaction between the polymer gelatin and graphene oxide resulting cross-linking and polymerization in a safe and tunable way intended for biomedical applications.

In the present study, we report that inert argon (Ar) microplasma treatment could be beneficial for modifying and reorganizing chemical groups in gelatin polymers for cross-linking and production of biomimetic nanocomposite gel-GO hydrogel system, which in turn would reduce the adversity of traditional chemical and other methods of cross-linking and polymerization (schematically illustrated as per Fig. 1).

The objectives of this work are to optimize, formulate, characterize, and evaluate gel-GO nano composite hydrogel system for its biocompatibility by systematic material characterization methods such as cross-linking index measurement, scanning electron microscopy (SEM), rheology, swelling behavior, Fourier transformation infrared (FTIR) spectroscopy, contact angle measurement, 3-[4,5-dimethylthiazol-2-yl]-2,5-diphenyl tetrazolium bromide (MTT) assay, microscopy, and a live/dead assay. Our study in resonance with Ar microplasma could be a useful tool for gel-GO nanocomposite hydrogel scaffold synthesis for tissue repair and regeneration. Further, it may prove to be a better approach for rendering an appropriate biomimetic scaffold designing platform for tissue engineering preventing chemical toxicity and related adverse effects.

## Materials and Methods

### Gelatin purification

Gelatin type B, isolated from bovine skin, was purchased from Sigma-Aldrich (St. Louis, MO, USA). Gelatin samples with an approximate isoelectric point of 5 and Bloom strength of 225 were used. Gelatin type-B powder was dissolved in distilled water for cross-linking the material to preserve the hydrogel structure. Various concentrations of gelatin solutions were prepared (7%, 8%, 9%, and 10%) in double-distilled water at 50 °C with continuous stirring for 30 min. Gelatin was purified to exploit the large number of functional side groups. We used acetone (Sigma-Aldrich, St. Louis, MO, USA) as a desolvating agent by combining it with the dissolved gelatin solution in this study in a ratio 1:1. The supernatant was discarded and the high-molecular-weight gelatin was re-dissolved by adding an equal volume of distilled water and stirred at 400 rpm at 40∼50 °C. The pH of the gelatin solution (5.7) was adjusted to near-neutral values of 7.4 in consideration of the biomimetic property of scaffold for body adaptability.

### Preparation of GO (high functionality)

Graphite (−325 mesh, 99.995% pure) microcrystalline powder was purchased from Alfa Aesar, USA. Potassium permanganate (KMnO_4_, 98%), Hydrogen peroxide (H_2_O_2_, 35%), and Ether [(C_2_H_5_)2O, 99 + %] were obtained from ACROS, Belgium. Potassium nitrate (KNO_3_, 95%) was purchased from JT-Baker (Center Valley, PA, USA). Hydrochloric acid (HCl, 37%) and Sulfuric acid (H_2_SO_4_ > 95%) were purchased from Scharlau, Spain. The graphene oxide used in this study was synthesized by a modified Hummer’s method. Similar details of the preparation can be found elsewhere (Wang et al., 2015). Briefly, 0.1 g of graphite was added in 10 mL of H_2_SO_4_ containing KNO_3_ (1 g) and magnetically stirred (300 rpm, 2 hrs) until a visually homogeneous dark gray solution formed. Then, KMnO_4_ (0.5 g) was slowly added to the previously formed solution and further stirred for 2 hrs at room temperature. After that, the solution mixture was put in water bath (IKA-HS7 digital; IKA Works, Staufen, Germany) at temperature 70 °C for 2 hrs. 200 g of composite precipitate mixture was removed from water bath, allowed to cool to room temperature by 350 g ice containing 5 ml of 35% H_2_O_2_ (to prevent precipitation of insoluble MnO_2_). Then, the mixture was centrifuged at 24,500 rpm for 30 min to get crude GO. The crude GO was then bath sonicated in 60 mL deionised water for 30 min followed by bath-sonication in 30 mL HCl and in 60 mL ether for 30 min. Finally, purified GO solid mixture was obtained by centrifugation of the dispersion mixture (24,500 rpm, 30 min) and was dried (Li et al., 2016; Kosynkin et al., 2009).

### GO characterizations

*Ex situ* characterization methods for natural graphite and as-produced GO were including X-ray diffraction (XRD), transmission electron microscopy (TEM), X-ray photoelectron spectroscopy (XPS), and micro Raman spectroscopy. For XRD, the dried GO powder was used as sample. The XRD was performed by BRUKER D2 PHASER- X-ray Powder Diffractometer (Bremen, Germany) (Cu Kα, λ = 1.54Å). For TEM, samples were prepared by dispersing GO in ethanol and then dropped onto 300 mesh holey lacy carbon grids on cupper support (Ted Pella, Inc., Redding, CA, USA) at ambient condition. The TEM images were observed by Hitachi H-9500 system, Japan. For XPS and Raman, the samples were prepared by dispersing GO in ethanol. Then thin sample films were prepared on Si wafer and dried in a hot air oven at 60 °C. XPS (VG ESCALAB 250; Thermo Fisher Scientific, Waltham, MA, USA) was performed using a monochromatic Al Kα X-ray radiation (10 kV and 10 mA). The source power was set to 72 W, and pass energies of 200 eV for survey scans and 50 eV for high-resolution scans. Raman scattering studies were performed at room temperature with a JASCO 5100 spectrometer (533 nm; JASCO, Tokyo, Japan).

### GO encapsulation into a gelatin matrix

High-functionality GO was weighed and grinded into a fine powder with a mortar and pestle. The finely powdered GO was added to distilled water and sonicated in ultrasonic water bath (Elmasonic P, 110 V, 720 W, 7 A; GmbH & Co, Weißenburg, Germany) for 30 min at 50 °C to obtain a uniformly dispersed solution. Dispersion of GO in biological media often requires surfactant stabilization or sonication to prevent aggregation (Ge et al., 2012). Various concentrations of gelatin-GO solutions were prepared by adding the GO solution drop-wise into different concentrations of previously melted purified gelatin solutions. For proper mixing and composite solution formation by covalent bonding between the GO and gelatin, the composite solutions were again sonicated for 1 h under above-described conditions. After sonication, we obtained uniformly dispersed GO in gelatin solution. The pH value was again measured and adjusted to a near-neutral value (7.4).

### Synthesis of gel-GO hydrogels by Ar microplasma

Argon microplasma was used here as a physical cross-linking tool for the gel-GO composite modification to form hydrogels. The anode is a platinum (Pt) foil (99.95%; Alfa Aesar, Ward Hill, MA, USA), which was immersed into the gel-GO solutions. The cathode is a stainless-steel capillary tube (with an inner diameter of 178 µm), which is also the gas inlet to create direct-current microplasma. Optimization of the argon microplasma process and gelatin and GO concentrations were the key factors in the entire process of gel-GO hydrogel fabrication along with various parameters including current, voltage, gas flow rate, conductivity, time of treatment, etc., were thoroughly investigated periodically to obtain the desired scaffold. The current and voltage are interdependent and affect the ionization of the plasma gas as well as free radical production (Bunshah & Deshpandey, 1985). It is important to maintain a steady current and voltage for uniform glow plasma discharge. Further, due to the conductive nature of gel-GO solutions, we used a resistor (300 W, 150 KΩ) and copper mesh as a barrier (to avoid surface burning) to stabilize the treatment process for the optimized microplasma conditions (2,500 V, 8.7 mA, 15 min, and a gas flow rate of 100 standard cm^3^/min). After plasma treatment, the resulting material gel-GO nanocomposite hydrogel was formed.

### Characterization of the gel-GO hydrogel

#### Degree of cross-linking

The degree of cross-linking was determined by a Ninhydrin assay, which is a direct way to determine the amount of free amino groups of untreated and plasma-treated gel-GO samples. The test sample was weighed and boiled with a Ninhydrin solution for 20 min. After that, the solution was cooled to room temperature, 95% ethanol was added, and the optical absorbance of the solution was recorded with a UV-visible spectrophotometer (Thermo Fisher Scientific, Rockford, IL, USA). At 570 nm using glycine at various concentrations as the standard. The amount of free amino groups in the gelatin before plasma treatment (Ci) and after (Cf) cross-linking is proportional to the optical absorbance of the solution. The degree of cross-linking of the various concentrations of gelatin were calculated as per Eq. (1). Results were the average of five independent measurements.

(1) \begin{eqnarray*}\text{Cross-linking index}(\text{%})=(\mathrm{Ci}-\mathrm{Cf})/\mathrm{Ci}\times 100\text{%}.\end{eqnarray*}

#### Surface morphology by SEM

Gel-GO hydrogels were prepared for SEM after lyophilization for 72 h. Small pieces of the hydrogel discs were cut off and mounted onto stubs using double-sided adhesive tape, and then gold-coated in a sputter coater (Hitachi E-1010; Tokyo, Japan) at 20 mA, 9 Å for 90 s. The cross-section morphologies of the gelatin discs were examined using a Hitachi S-3500 SEM with an accelerating voltage of 15 kV. Fifteen different pores were randomly selected, and the average pore diameters were calculated. Results of five independent runs were averaged.

#### Spectral change observation by FTIR spectroscopy

The FTIR spectra for all samples were obtained in KBr pellets using a Perkin-Elmer Precisely-FTIR spectrophotometer (Melville, NY, USA) in transmission mode at a wavelength range of 400∼4,000 cm-1

#### Gel-GO nanocomposite visualization by confocal microscopy

For three-dimensional, high-resolution, non-destructive imaging of the gel after treatment, confocal microscopy was used to visualize the colloidal structure of the gel-GO composite. Confocal microscopy offers several advantages over conventional wide-field optical microscopy, including the ability to control the depth of field, reduction or elimination of background information away from the focal plane (that leads to image degradation), and the capability to collect serial optical sections from thick specimens. Furthermore, confocal microscopy X–Z plane imaging was used to analyze morphological changes of the gelatin matrix with or without GO. Test samples were evaluated on a confocal dish using confocal laser scanning microscopy {MitoTracker Red 580 (Invitrogen [Molecular Probes], Gibco, Carlsbad, CA, USA} to check the structural changes.

#### Swelling behavior

To study the swelling behavior, untreated gel-GO nanocomposites and microplasma-treated hydrogels were immersed in deionized water at 37 °C. Samples were taken out from deionized water at selected time intervals, wiped with tissue paper to remove surface water droplets, and weighed further. Wet and dry weights were measured and considered to evaluate the swelling ratio.

The swelling ratio (*S*) was calculated using the following equation:

(2) \begin{eqnarray*}S=({W}_{t}/{W}_{0});\end{eqnarray*}

where, *W*_*t*_ is the weight of the swollen sample at a certain time point, and *W*_0_ is the initial weight of the sample.

#### Rheological analyses

All rheological experiments were conducted using a Thermo Scientific™ HAAKE RheoStress 1 rotational cone plate rheometer with an angular resolution of 300 nanorad and a low-inertia drag cup motor. The plate diameter used was 15 mm. The sample was placed on horizontal plate and a shallow cone placed into it. The angle between the surface of the cone and the plate was around 1 degree. The storage modulus (G′) and loss modulus (G″) were evaluated for gelatin w/o GO, untreated and plasma-treated gel-GO hydrogel samples in a temperature range of 7∼60 °C to check the gel strength at general physiological conditions specifically at 37 °C. The viscoelasticity was observed at a frequency 1 Hz for up to 3,000 s. The sol-to-gel and gel-to-sol state changes were correlated with an ideal biomimetic scaffold for orthopedics and specifically for soft tissue engineering fields with tunable property.

#### Water contact angle for hydrogel hydrophilicity

Water contact angles of untreated and microplasma-treated hydrogels were measured using the DIGIDROP-GBX contact angle measurement system (Bourg-de-Peage, France). The sample materials were cut into pieces (*n* = 3), then deionized water droplet was gently deposited on each sample through a micro-syringe, images were captured for up to 30 s after the water droplet was dropped on the surface of the material, and the average contact angle was measured.

#### 3-[4, 5-Dimethylthiazol-2-yl]-2, 5-diphenyl tetrazolium bromide (MTT) assay of cell proliferation

The MTT cytotoxicity assay is a standard method prior to detailed *in vitro* study. Equal sizes, weights, and volumes (5 mm × 5 mm × 5 mm), (0.3 g), (300 µL)) of untreated and treated hydrogel scaffold materials in triplicate were incubated in 2 mL Dulbecco’s modified Eagle’s medium (DMEM; Gibco, Sigma-Aldrich) with 10% v/v of fetal bovine serum in 24 well cell culture plates at 37 °C, in 5% CO_2_ and fully humidified air for 1, 3, 5, 7, 10, and 14 days. The liquid extraction medium (10 µL each) were collected for MTT assay. Here, we used an indirect method of measurement to assess cell viability. The L929 mouse fibroblast cell line (ATCC (NCTC clone 929; L cell, L-929 derivative of Strain L; ATCC CCL-1)) with an initial density of 5,000 cells/cm^2^ per well at a volume 100 µL were seeded in 96-well plates and were cultured for 24–48 h for proper attachment and growth. Then, periodically collected liquid extracts (10 µL each) were added to each well and incubated for 24 h. Finally, the cell metabolic activity was determined using a MTT cell proliferation kit (Sigma-Aldrich, Gibco, St. Louis, MO, USA). Further, the microplsma treated hydrogel system was compared with traditional genipin crosslinked gelatin hydrogel as control group for cytotocity assay by MTT assay with same experimental conditions as mentioned previously for untreated and microplasma treated gelatin hydrogel.

#### Microscopy to observe cellular and hydrogel interactions

Microscopic surface images of MG-63 cells seeded on various samples of gels and hydrogels for 24 h were observed under an inverted microscope (Nikon Eclipse TE2000-U Inverted Microscope) to clearly visualize cellular growth and proliferation as direct method for primary biocompatibility study.

#### Live/dead assay

The viability of cells on the surface of the gel-GO was assessed by live/dead staining. In brief, MG-63 cells were seeded directly onto the surface of gelatin, microplasma-treated gelatin, untreated gel-GO composite gels, and microplasma-treated gel-GO hydrogels in 6-well plates. After 24 h of incubation, cells/specimens were rinsed three times with phosphate-buffered saline (PBS). The cell laden hydrogels were cryosectioned. The slides were then incubated with live/dead stain (2 mM calcein AM and 4 mM ethidium homodimer-1) (Thermo Fisher Scientific, Dreieich, Germany) for 30 min at room temperature (RT). Viable cells (green) and dead cells (red) were counted under a fluorescence microscope (Olympus IX70, Tokyo, Japan).

**Figure 2:**
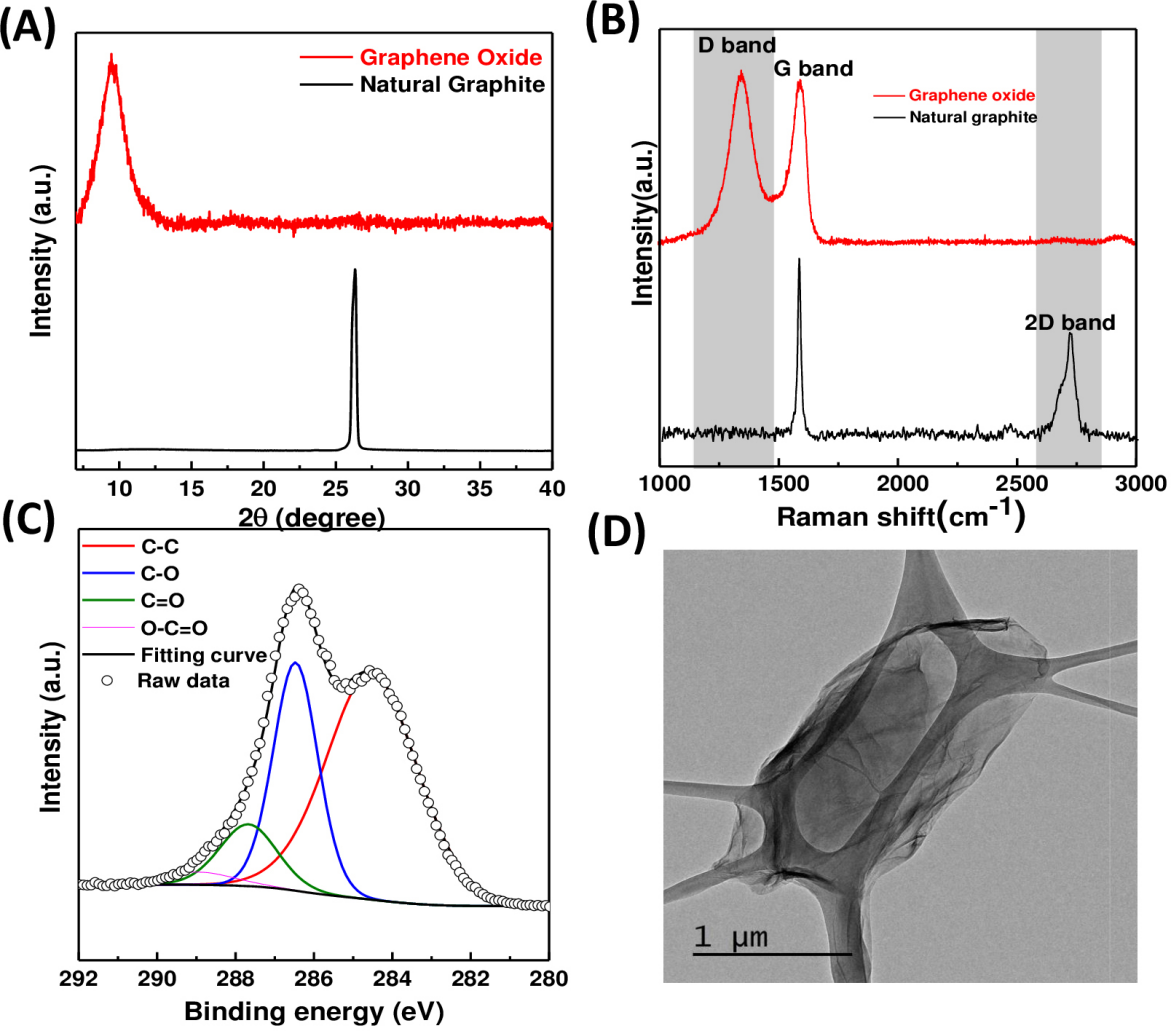
XRD, Raman, XPS and TEM for GO characterization. (A) XRD patterns of natural graphite and as-produced GO. (B) Micro Raman spectra of natural graphite and as-produced GO. (C) C1s peak of GO in the HRXPS spectrum. C1s peak was deconvoluted to C–C, C–O, C=O, and COOH surface functionalities at 284.4, 286, 287, and 289 eV, respectively. (D) TEM image of GO.

#### Statistical analysis

Statistical analyses of all correlated data along with the MTT cytotoxic assay of the untreated gel-GO and microplasma-treated gel-GO hydrogel samples were analysed with the help of Graph pad prism software preferably by a two-way analysis of variance (ANOVA). Each experiment was independently performed and duplicated. Differences were considered significant at the *p* < 0.05 level.

## Results

### GO characterization

After GO synthesis, it was charactrerized by XRD, Raman, XPS and TEM as shown in Fig. 2. The microstructure of natural graphite and the as-produced GO was obtained from the XRD characterization. In the XRD patterns, the natural graphite showed a peak at ∼26.3°(Fig. 2A), attributed to the (002) plane of the interplanar graphite with a d spacing of 0.34 nm according to the Bragg’s Law (Futaba et al., 2011). For the GO, a characteristic peak of oxidized GO structure at ∼9.4°was observed (Kosynkin et al., 2009), suggesting the existence of a mixture of graphite and oxidized GO. The calculated d spacing was increased, which was due to the functional groups generated between the adjacent layers of as-produced GO during the process of oxidation. Further structural information of the as-produced GO was obtained from micro Raman spectroscopic characterization. As shown in Fig. 2B, the representative Raman spectra of natural graphite and as-produced GO. Three bands were observed around 1,349 cm^−1^, 1,596 cm^−1^, and 2,679 cm^−1^, which were respectively assigned to the D-band, G-band, and 2D-band of carbon. The surface functionalities of the as-produced GO were further studied by XPS. Figure 2C and the C1s peaks of HRXPS spectra of natural graphite and as-produced GO were found to be deconvoluted to several peaks at 284.4, 286, 287, and 289 eV, corresponding to sp^2^ C–C, C–O, C=O, and COOH surface functionalities respectively. The results suggested that the as-produced GO possessed oxygen-containing functional groups in agreement with previous XRD and Raman results (Cataldo et al., 2010; Higginbotham et al., 2010). Further, the topological TEM image of high functionality graphene oxide was shown in Fig. 2D.

**Figure 3:**
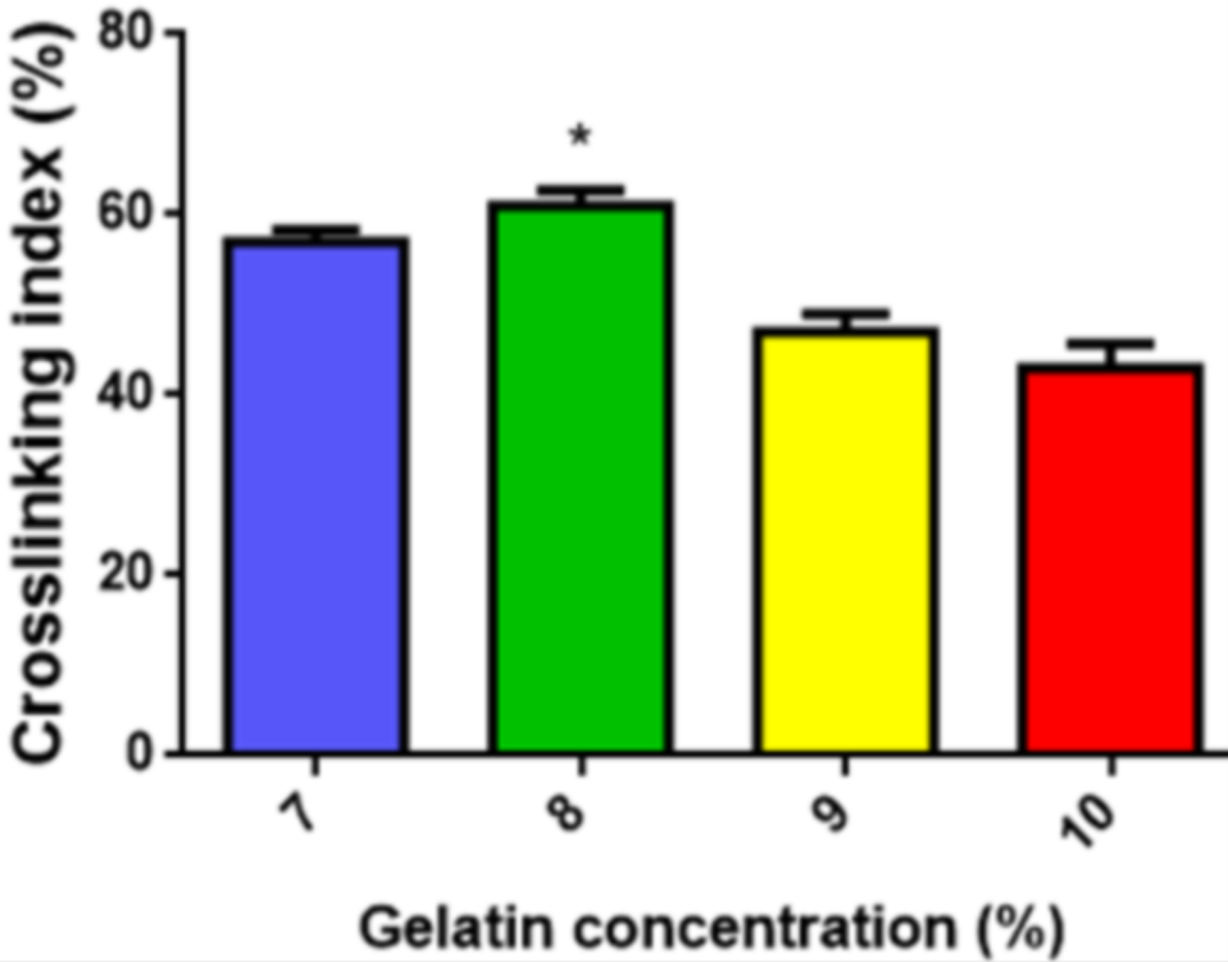
Cross-linking index. Cross-linking index of various concentrations of purified gelatin. An asterisk indicates statistically significant difference of 8% purified gelatin group cross-linking (^∗^*P* > 0.05 and *n* = 5) as compared with other groups (7%, 9%, 10%).

### Gel-GO hydrogel characterization

#### Cross-linking index/degree of cross-linking

A cross-linking index measurement here was used as an analytical tool to survey additional modifications to the initial polymer gelatin. Here, 8% purified gelatin during microplasma treatment achieved the highest degree of cross-linking of 61 ± 2% (Fig. 3). Thus, in view of the uniform distribution of plasma energy into the composite solution and to avoid surface blocking and burning of a dense solution, 8% purified gelatin was selected with the optimal viscosity and density suitable for the desired gel-GO nanocomposite tissue engineering scaffold fabrication.

#### Morphology and pore size analysis

**Figure 4:**
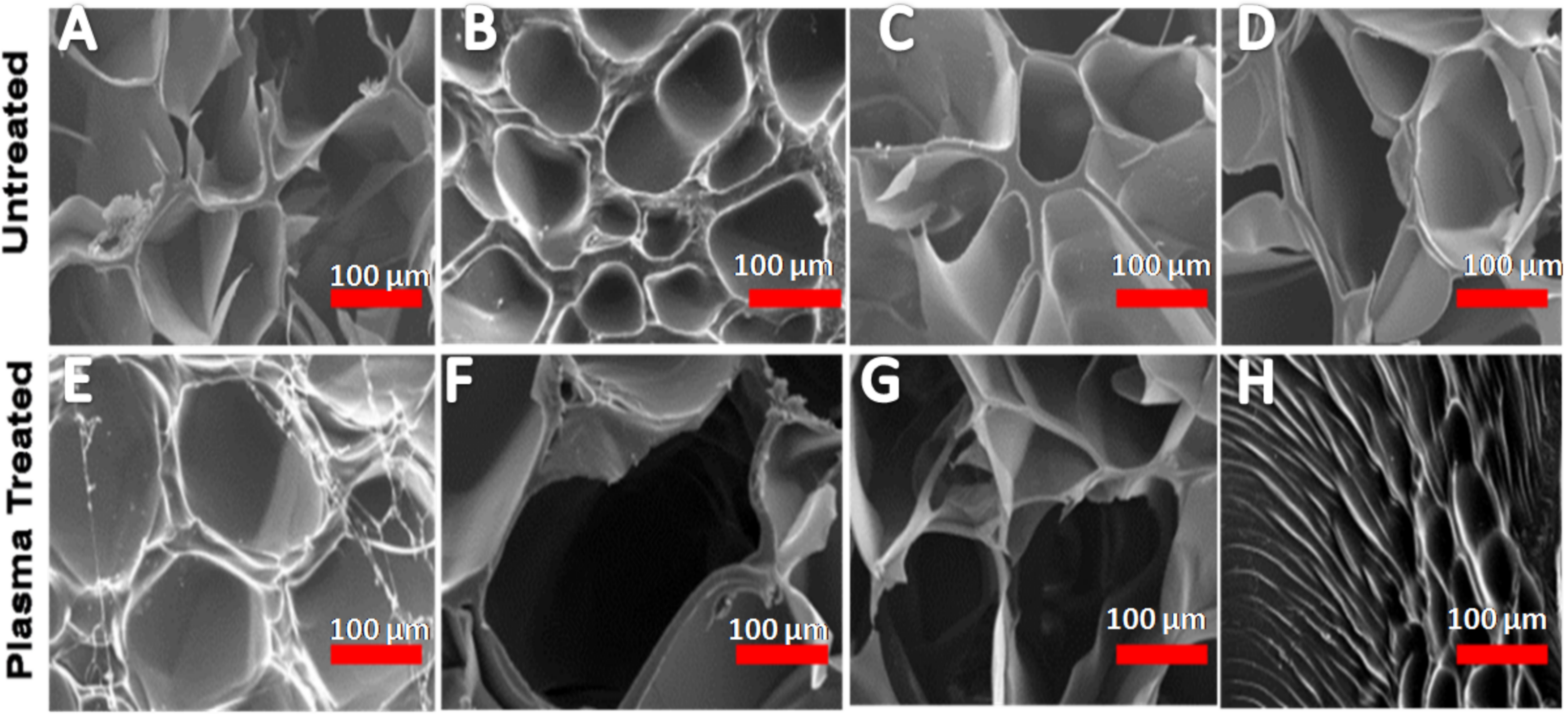
Scanning electron microscopy. Morphological observations and representative scanning electron microscopic images of various concentrations of gelatin—graphene oxide nanocomposite material before (A) 7%, (B) 8%, (C) 9%, (D) 10% and after cross-linking (E) 7%, (F) 8%, (G) 9%, (H) 10% showing 8% purified gelatin achieving highest pore size 287 ± 27 µm after microplasma treatment. Scale bars: 100 µm.

**Figure 5:**
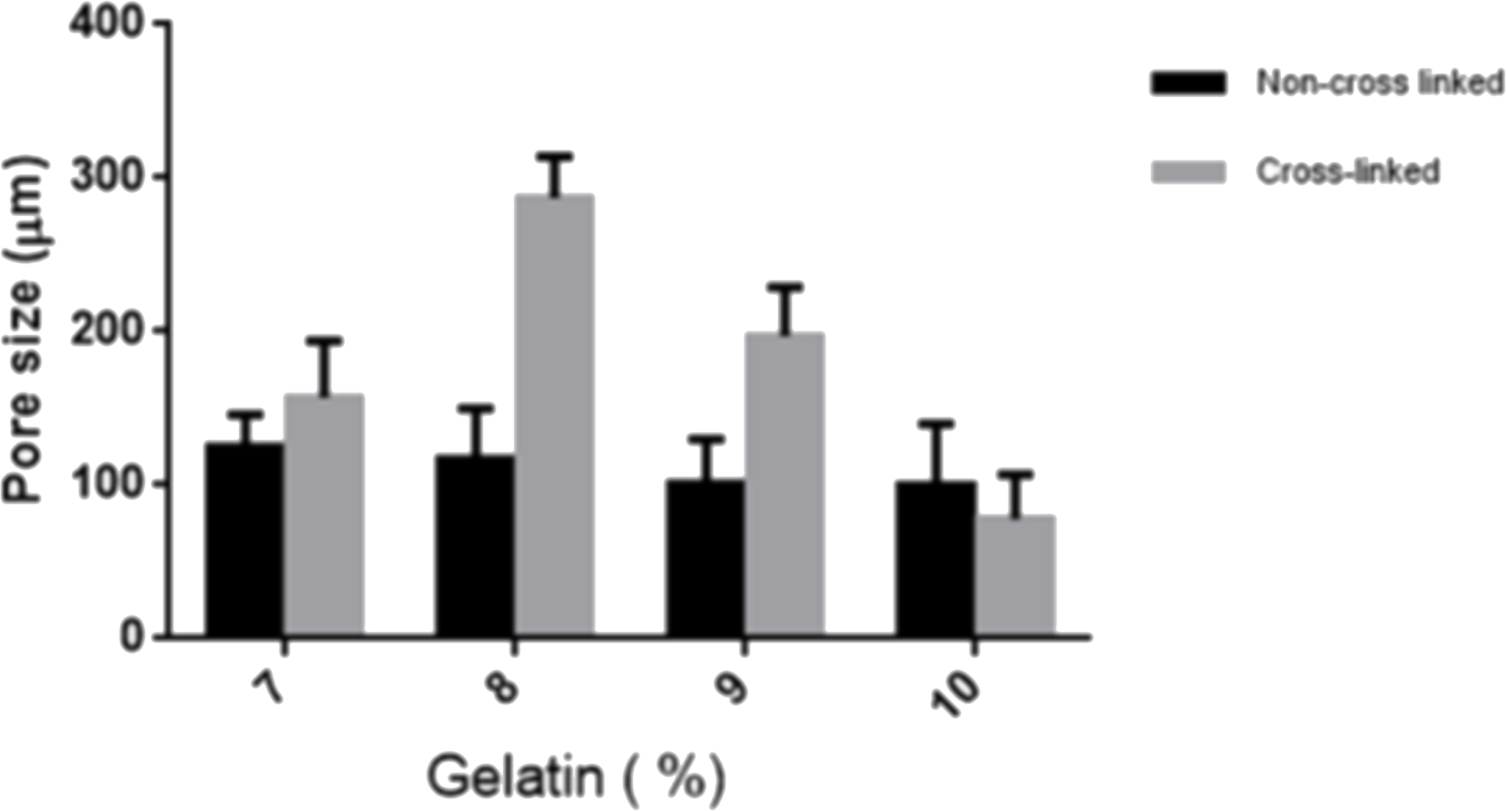
Pore size. SEM characterization of porous structure of Gelatin. Pore size of various concentrations of gelatin- graphene oxide nano composite before and after Ar-microplasma treatment. An asterisk indicates statistically significant differences (^∗^*P* < 0.05; *n* = 5) between the non-cross-linked and cross-linked groups for each concentration of gelatin groups.

Detailed images of the morphology of the samples were observed by SEM. As per SEM images, the pore size of the 8% purified gel-GO hydrogel formed after plasma treatment was found to be better 287 ± 27 µm (Figs. 4 and 5) among all the groups, adequate for cellular penetration and proliferation.

**Figure 6:**
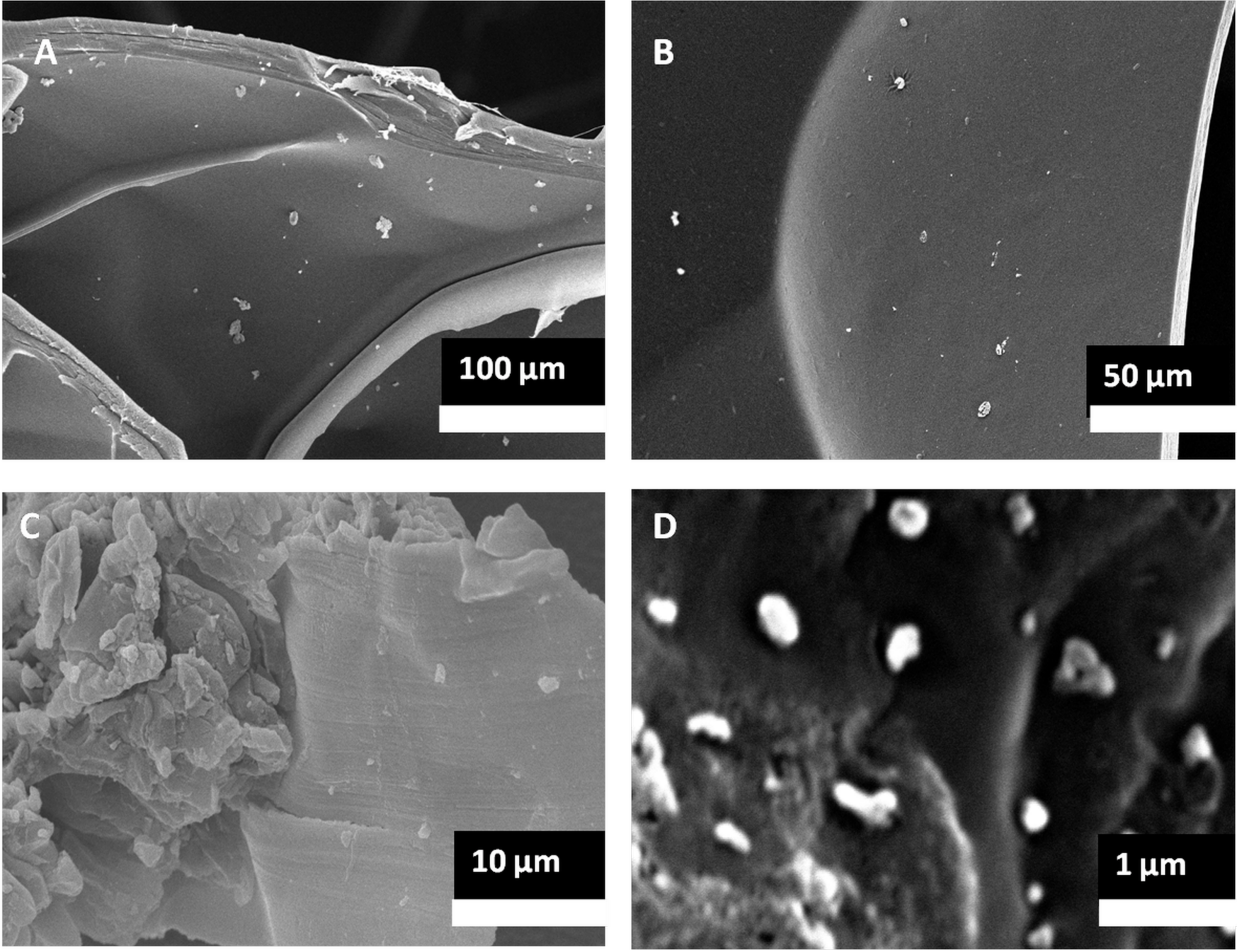
SEM of gel-GO nano composite. Scanning electron micrographic images of plasma treated nano graphene oxide encapsulated in gelatin matrix observed at various magnifications (lower to higher magnification from A to D).

Further, in Fig. 6, GO encapsulation was clearly visualized in the gel-GO nano composite hydrogel matrix system. The SEM images showed that the graphene oxide nano particles were embedded in the polymeric gelatin matrix system.

#### FTIR Analysis

**Figure 7:**
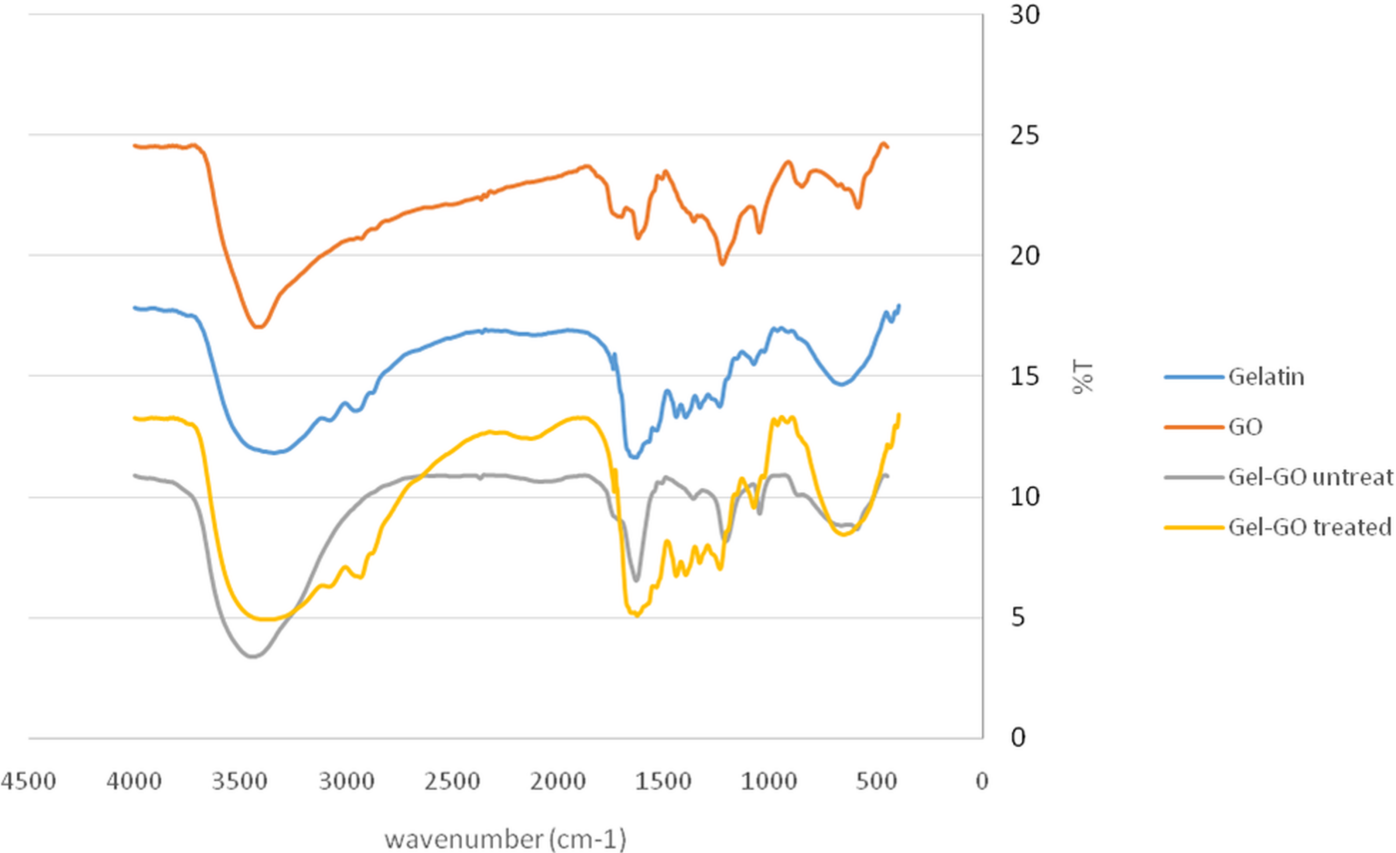
FTIR spectra analysis. FTIR spectra of: original high functionality graphene oxide (red line), gelatin (blue line) untreated gelatin-graphene oxide gel sample (gray line) and Ar- microplasma treated gelatin-graphene oxide cross-linked sample (hydrogel) (yellow line). The FTIR spectrum of the un-cross-linked gelatin and graphene oxide revealed a structure that was quite similar to raw gelatin and graphene oxide after gelatine grapheme oxide nano-composite hydrogel preparation.

FTIR spectroscopy offers a vast assay of analytical tools. Different spectral changes during cross-linking and modification were observed by FTIR spectroscopy in our study. The FTIR spectrum of GO is shown in Fig. 7, and the appearance of characteristic stretching and bending vibrations confirms the presence of various functional groups in the structure of GO. Bands at 1,053 and 1,366 cm^−1^ correspond to stretching vibrations of C–O bonds. The intense band at 1,227 cm^−1^ is due to stretching vibration of epoxy C–O bonds. The C=C (aromatic) and carbonyl stretching bonds were observed at 1,627 and 1,707 cm^−1^, respectively. A broad band at 3,415 cm^−1^ can be attributed to O–H stretching vibrations.The FTIR spectrum of gelatin shows the presence of C=O stretching vibrations of amide I at 1,639 cm^−1^, N–H bending of amide II at 1,514.2 cm^−1^ and amide III at 1,211.8 cm^−1^, respectively, along with an N–H stretching vibrational band due to a primary amine at 3,442.4 cm^−1^. After incorporation of gelatin into GO, there was a decrease in the intensity and shift in the vibrational frequency of C=O stretching of GO which could be attributed to the possible formation of an ammonium carboxylate complex through protonated amino groups of gelatin and carboxyl groups of GO. The merging of the vibrational frequency of amide bonds of gelatin and aromatic C=C vibrational stretching of GO led to a highly intense band at 1,632 cm^−1^. A shift in the stretching vibration of amide III of gelation at 1,211.8–1,241.5 cm^−1^ was also observed. The FTIR of microplasma treated gelatin-graphene oxide showed that, the chemical architecture of gelatin and graphene oxide assumed to be linked via electrostatic interactions and some amide bonds was intact as no drastic changes in the vibrational frequencies of the key functional groups were observed. The intact functional groups are highly desired so as to prevent the loss of biomedical applicability of the fabricated hydrogel in case of significant changes in the structure.

#### Confocal microscopy for gel-GO composite matrix

**Figure 8:**
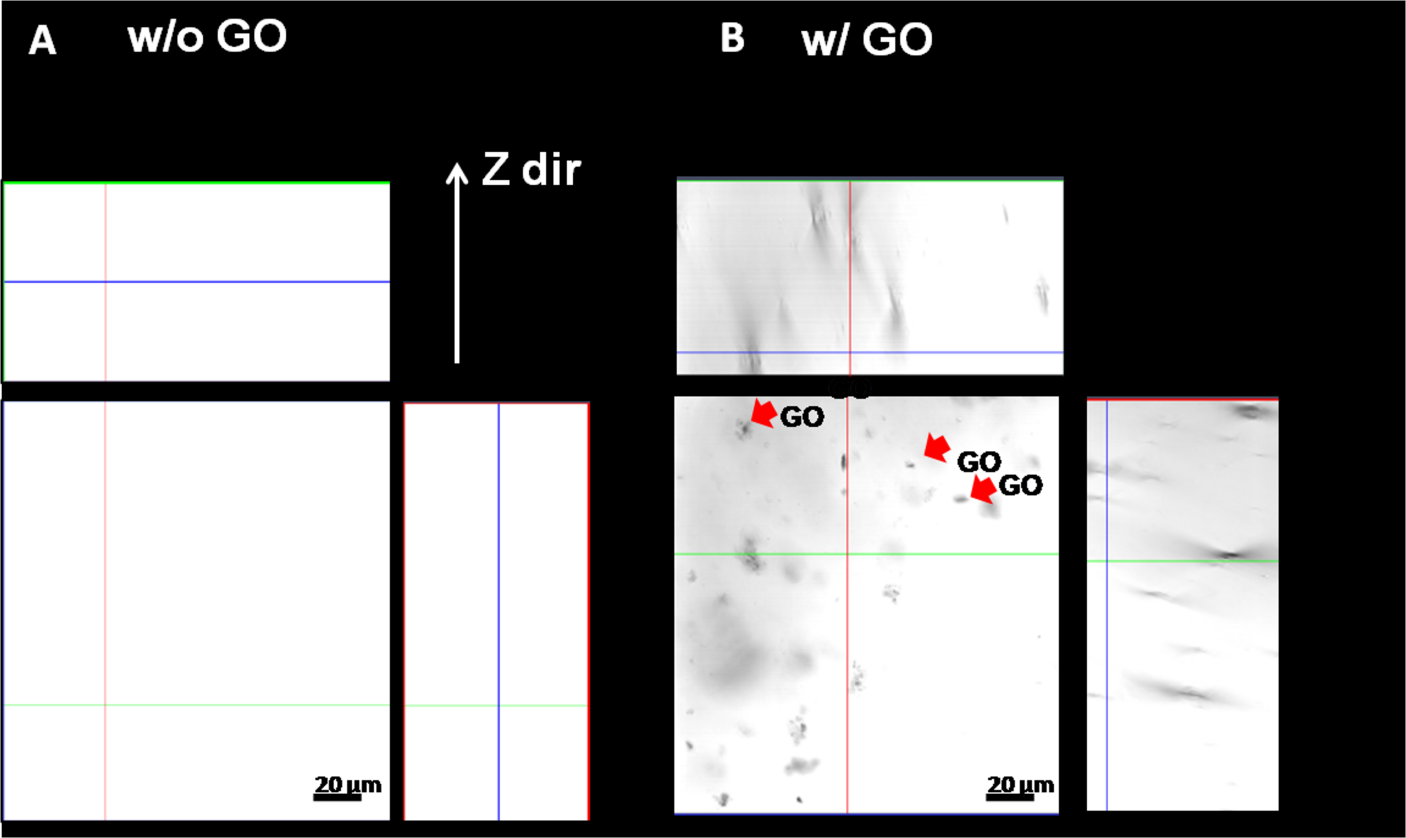
Confocal microscopy. (A) gelatin w/o GO and (B) gelatin w/ GO nano composite hydrogel system (X–Z plane imaging) indicating graphene oxide (indicated by red arrows) encapsulated in gelatin matrix system (scale bar 20 µm).

Confocal microscopy revealed the true 3D resolution X–Z plane images of samples. It can be observed from Fig. 8 that the gelatin matrix without GO appeared flat and transparent in distinct layers. It was further elucidated using the technology of differential interference contrast from the confocal microscope. In contrast, the gel-GO group showed GO embedded as a crystal-dot-like structure within the gelatin matrix.

#### Swelling properties for degradation

**Figure 9:**
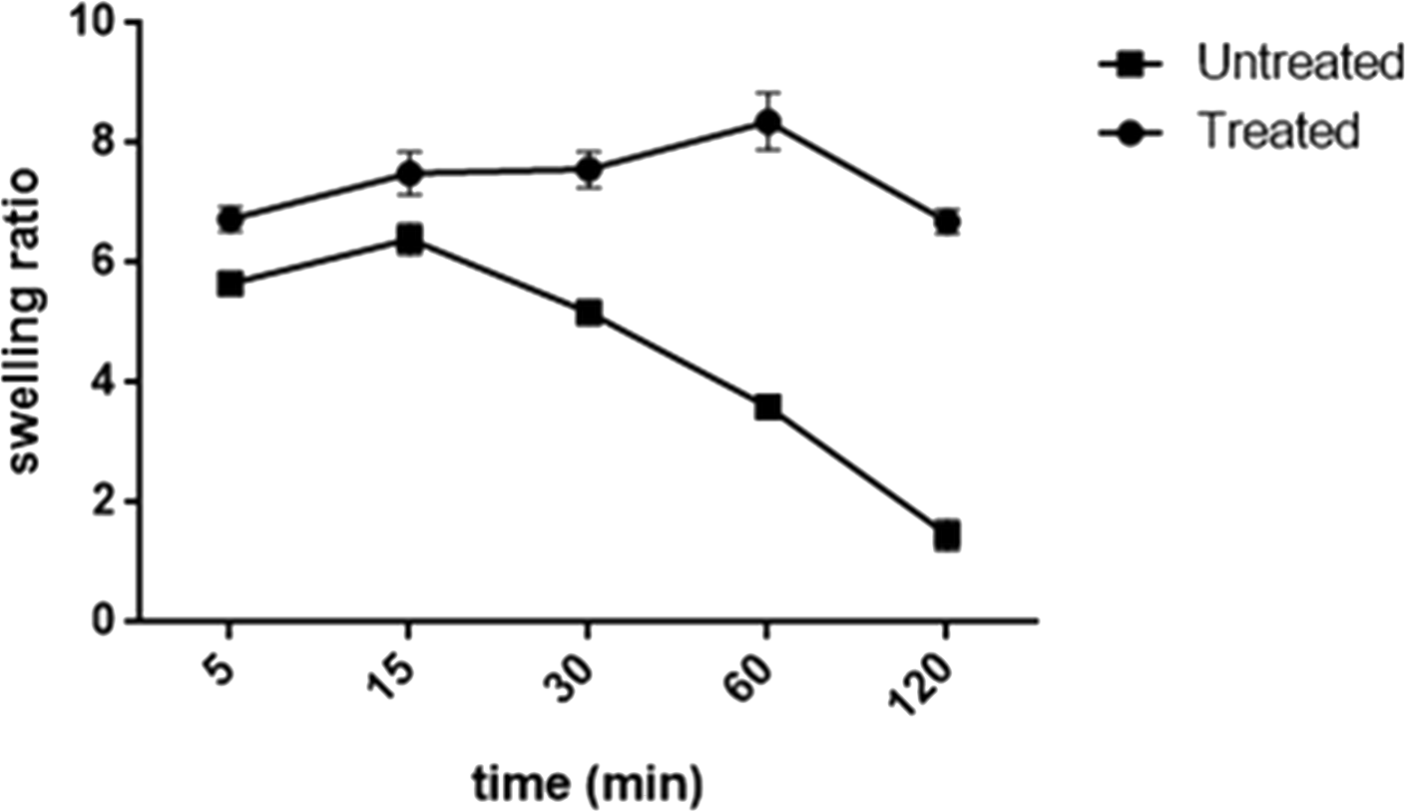
Swelling property. The swelling property of untreated Gel-GO matrix and microplasma treated Gel-GO nanocomposite hydrogels in deionized water (pH 7.4 and temp. 37 °C) showing significantly betterswelling property, stability and visco-elasticity of microplasma treated Gel-GO hydrogel in comparison to untreated Gel-Go material (*n* = 3).

The degree of cross-linking, solvation, and degradation were primarily measured through the swelling behavior. The water-absorption capability was evaluated by monitoring the swelling ratio of untreated and treated gel-GO composite samples as a function of time. As shown in Fig. 9, the two-way ANOVA revealed a significant effect of microplasma treatment and its interactive effect on the swelling properties of the gel-GO nanocomposite hydrogels. Untreated samples exhibited swelling ratios of 5.6∼6.4 within 15 min, and showing disintegration into fragments by water uptake with a swelling ratio of 1.4 within 120 min. In plasma-treated hydrogel groups, the swelling ratio reached a level 8.3 from 6.5 within 1 h and then equaled the initial value of 6.5 in 2 h of incubation in deionized water at 37 °C (*p* < 0.05).

#### Rheology for gel property analyses at body temperature

**Figure 10:**
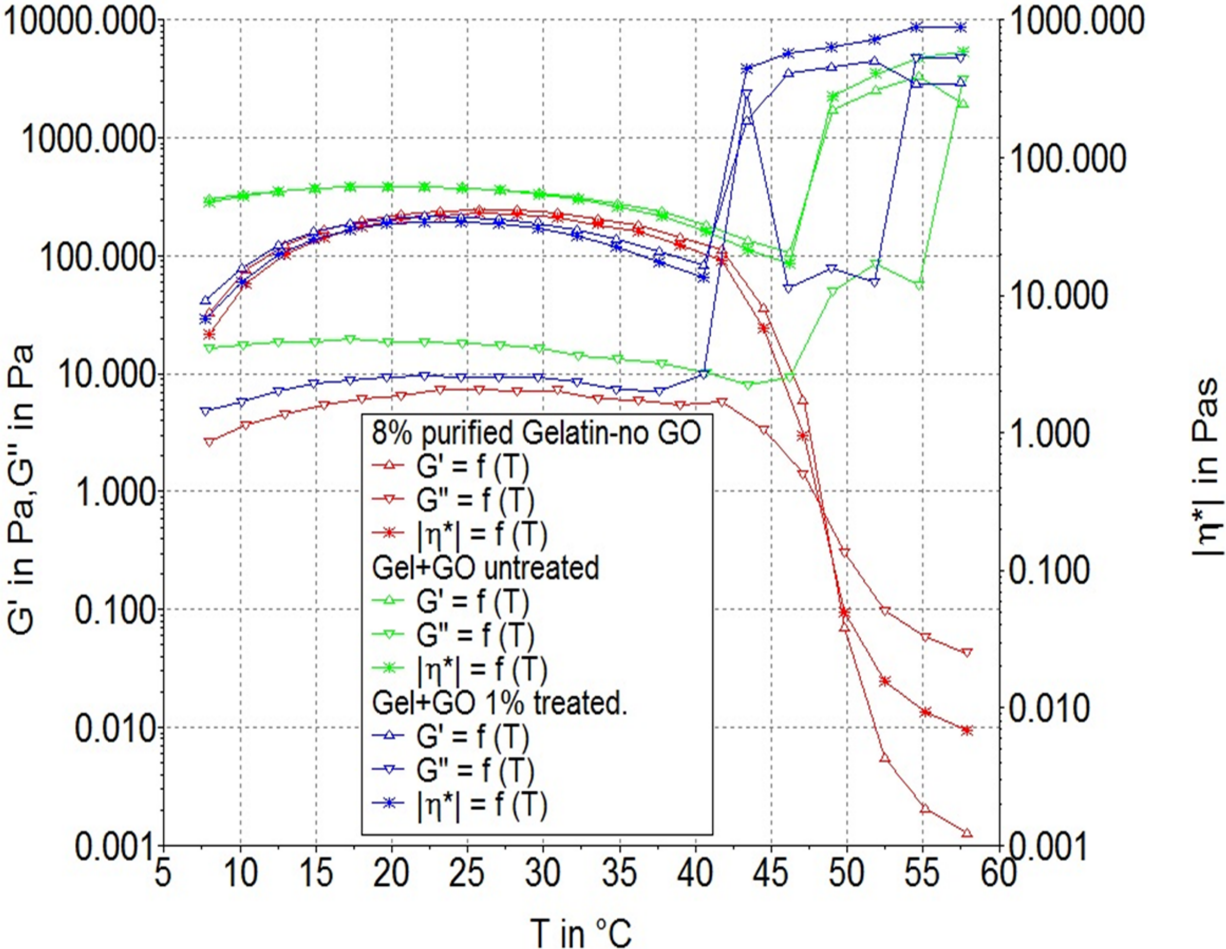
Rheology. Rheological tests for Storage modulus: G′ (G prime), Loss modulus, *G*″ (G double prime) of gelatin without GO (red curve), gelatin-graphene oxide nano composite samples before plasma treatment (green curve) and after microplasma treatment (blue curve) at various temperature conditions indicating the differences between gelatine w/o GO, non-cross-linked and cross-linked groups.

Modifications of *in situ* gelling were measured by rheometry. The rheological study mainly focused on the storage modulus (G′) and loss modulus (G″) at a specific frequency (1 Hz) and temperature range of (7∼60 °C) with particular focus at 37 °C which is the same as the body’s physiological temperature. For analytical purposes, if *G*′ > G″, then the material is more solid than liquid, and it maintains a gel state. The resulting gel-GO nano composite hydrogel was found to be with better and adequate gel strength (as shown in Fig. 10) in comparison to only gelatin w/o graphene oxide and untreated gel-GO nano composite. Interestingly in the case of microplasma-treated gel-GO samples, the storage modulus increased by 50% more than that of the untreated group, which was found to be even more than that of gelatin w/o GO at 40 °C temperature resulting in increased viscosity and gel strength properties (Table 1).

**Table 1:** Rheology. Showing storage modulus: G′ (G prime) > Loss modulus, G″ (G double prime) up to temperature 40 °C in case of microplasma treated Gel-GO nanocomposite hydrogel with better visco-elasticity in comparison to untreated Gel-GO and gelatine w/o GO samples (*n* = 5).

Temp. (° C)	Gel w/o GO		Untreated		Treated	
	G′	G″	G′	G″	G′	G″
5	32 ± 2	2 ± 3	42 ± 3	4 ± 0.5	299 ± 6	16 ± 3
10	76 ± 3	3 ± 3	78 ± 4	6 ± 0.3	329 ± 3	17 ± 4
20	198 ± 3	6 ± 1	205 ± 4	9 ± 0.5	386 ± 7	18 ± 2
30	177 ± 1	6 ± 3	189 ± 5	9 ± 0.5	335 ± 5	16 ± 3
37	104 ± 3	7 ± 3	108 ± 4	7 ± 0.3	236 ± 7	12 ± 3
40	79 ± 3	5 ± 2	83 ± 3	10 ± 0.7	182 ± 5	10 ± 1

#### Water contact angle for wettability and hydrophilicity

**Figure 11:**
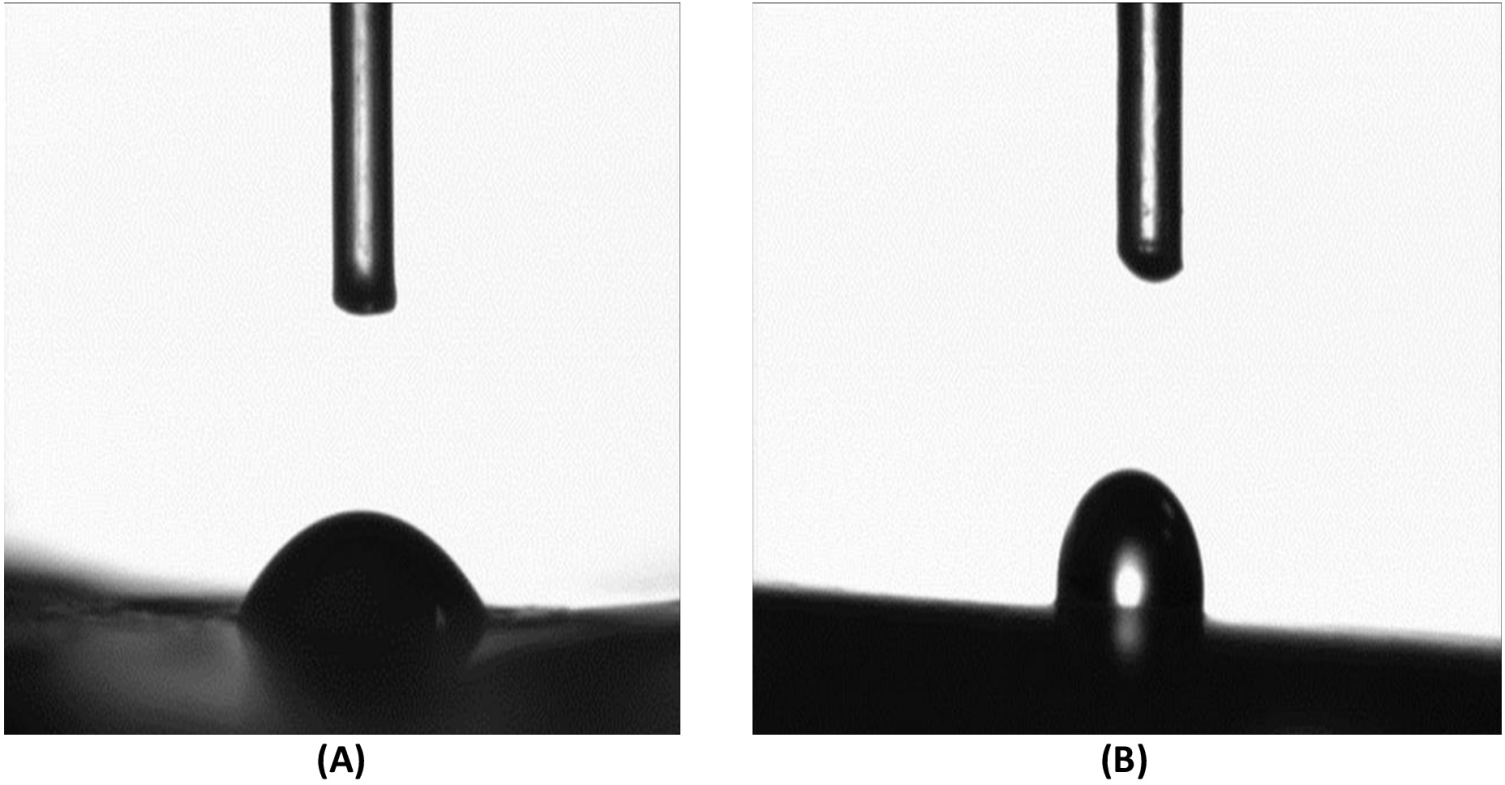
Water contact angle. Water contact angle measurements of untreated Gel-GO nano composite material (A) and microplasma treated Gel-GO hydrogel (B) (*n* = 5) showing decreased in hydrophilicity due to increase in water contact angle from 49° ± 7.8°to 78° ± 3.7°.

**Figure 12:**
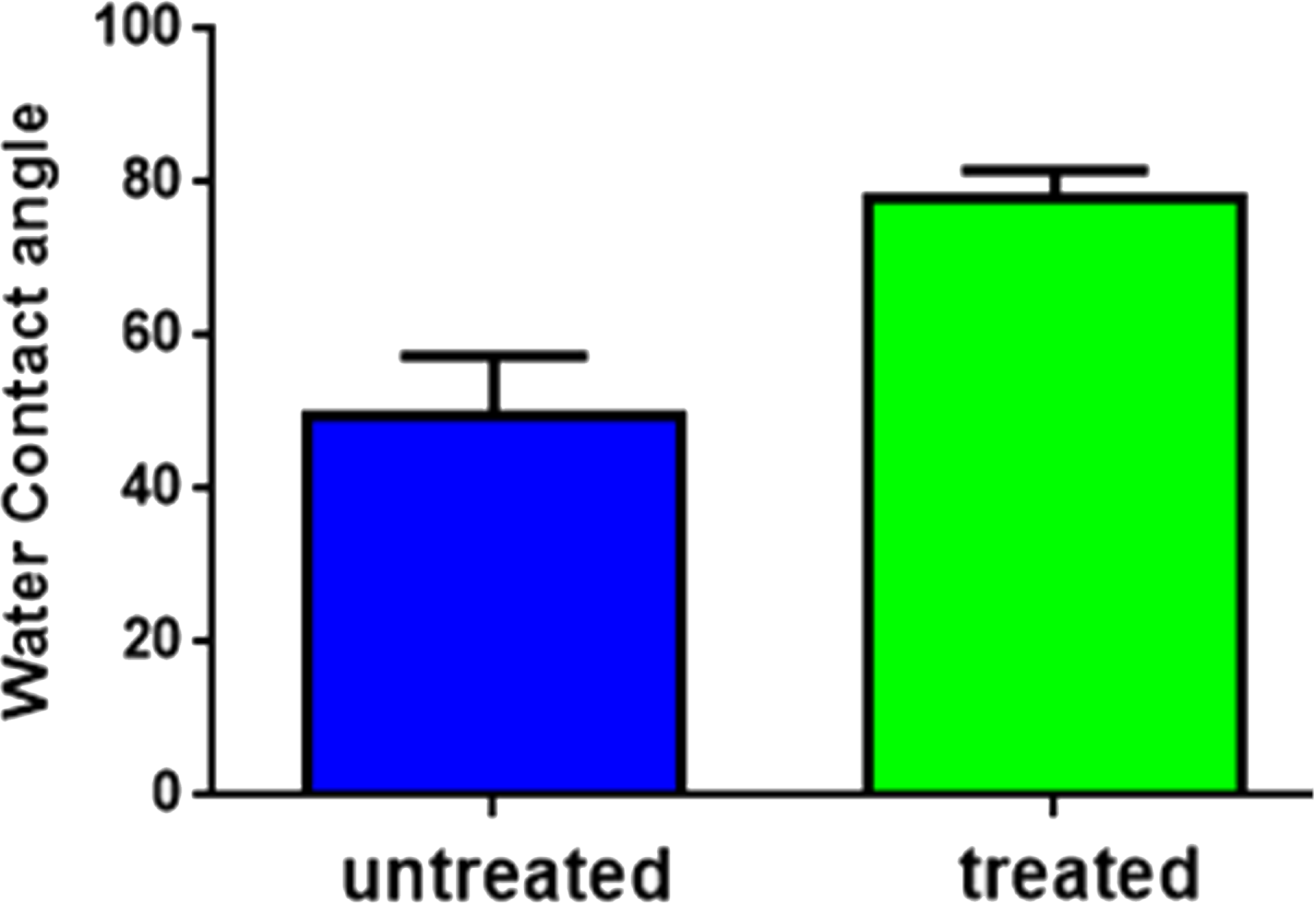
Water contact angle (graphical). Water contact angle measurements of untreated Gel-GO nano composite material and microplasma treated Gel-GO hydrogel (*n* = 5).

The water contact angle measurement is the inverse measurement procedure to know the wettability, hydrophilicity, and hydrophobicity. The water contact angles for both untreated and plasma-treated samples revealed that both were in the hydrophilic range of <90 ° (Figs. 11A and 11B). However, after plasma treatment, the water contact angle increased from 49°(untreated) to 78°(treated) as observed in Fig. 12.

#### MTT assay

**Figure 13:**
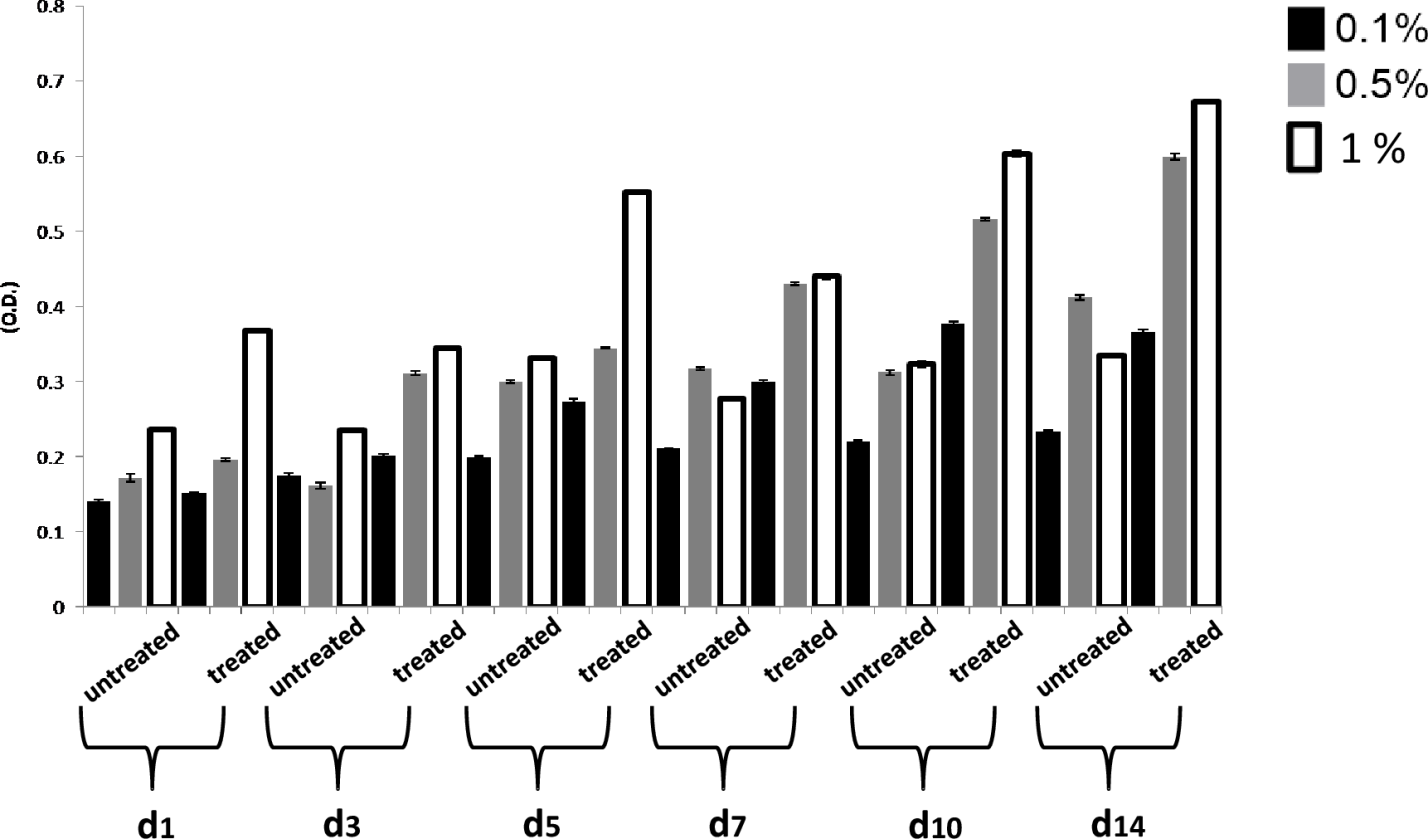
MTT assay (gel-GO untreated and gel-GO treated). Indirect MTT analysis of (L929 fibroblast) untreated and microplasma treated Gel-GO hydrogel (various weight percentage of graphene oxide in gelatine) after 1, 3 and 5, 7, 10 and 14 days of incubation with DMEM. MTT assay revealed the highest cell viability for microplasma treated Gel-GO hydrogels with 1 wt% of graphene oxide (*n* = 6).

Metabolic activities of L929 cells were assessed in an indirect cytotoxicity test according to ISO 10993-5 guidelines before going to detailed *in vitro* cell specific studies. Cytotoxicity results were analyzed periodically from day 1 to maximum 14 days as shown graphically in Fig. 13 for GO and its effect on cell viability. At the intermediate 0.5 wt% GO concentration, there was less-profound toxicity toward cells, while increases in cell viability and proliferation were observed at a concentration of 1 wt% GO along with gelatin.

**Figure 14:**
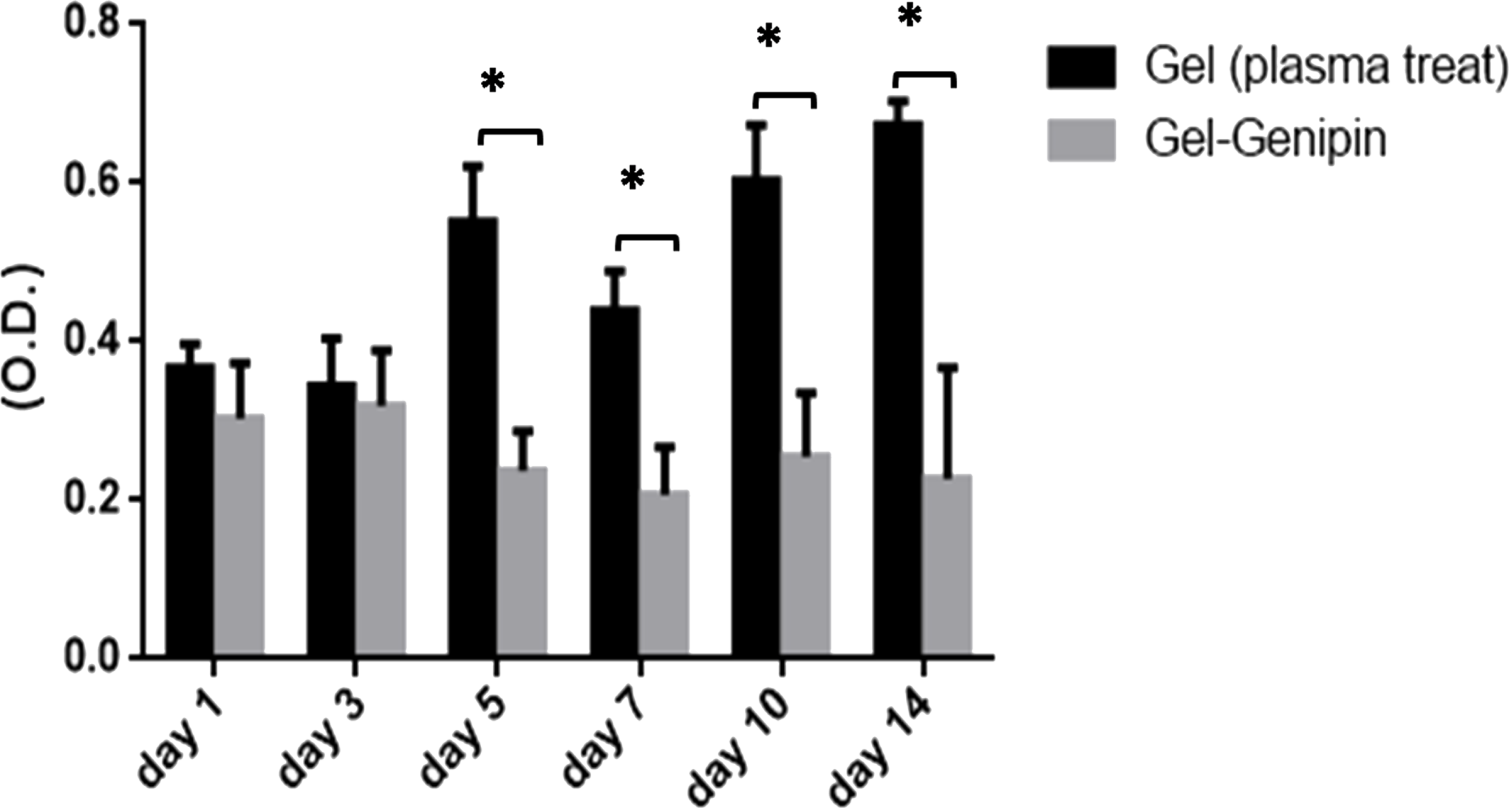
MTT assay (plasma treated hydrogel and genipin treated hydrogel). Comparative MTT analysis of microplasma treated gelatin hydrogel and genipin cross-linked gelatin hydrogel by collection of liquid extraction medium at 1, 3 and 5, 7, 10 and 14 days of incubation with DMEM. MTT assay (L929 cell line) revealed the highest cell viability for microplasma treated gelatin hydrogel (^∗^*P* < 0.05; *n* = 6).

**Figure 15:**
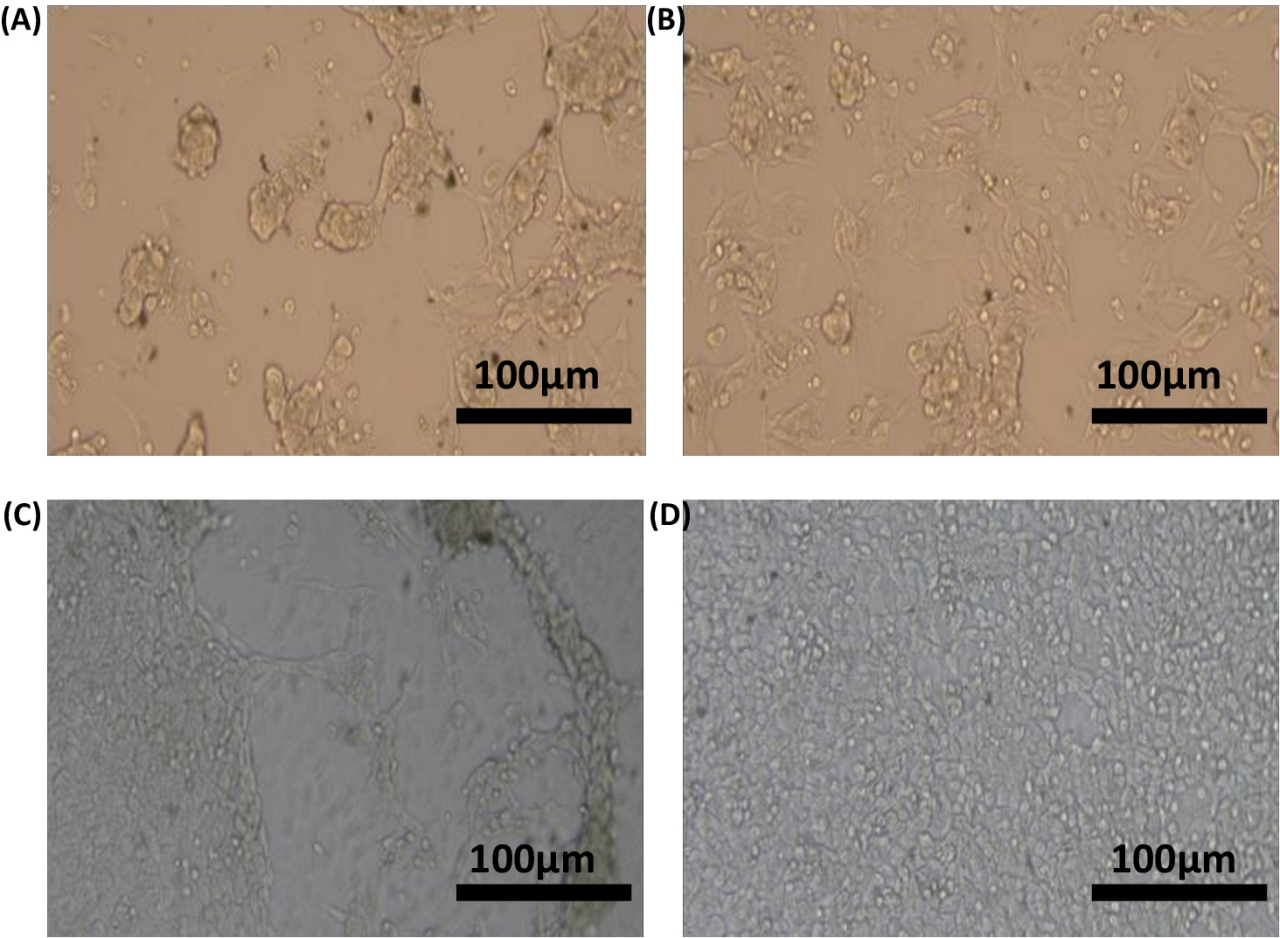
Inverted microscopy. Inverted microscopy of untreated and microplasma treated gelatin–graphene oxide hydrogel seeded by osteosarcoma cell lines MG63 at 24 h. Effect of gelatin untreated (A) and gelatin microplasma treated (B) and gelatin-graphene oxide untreated (C) and microplasma treated (D) on MG63 cell lines and its proliferation was found comparatively with better result in microplasma treated gelatin—graphene hydrogel group (scale bar 100 µm).

**Figure 16:**
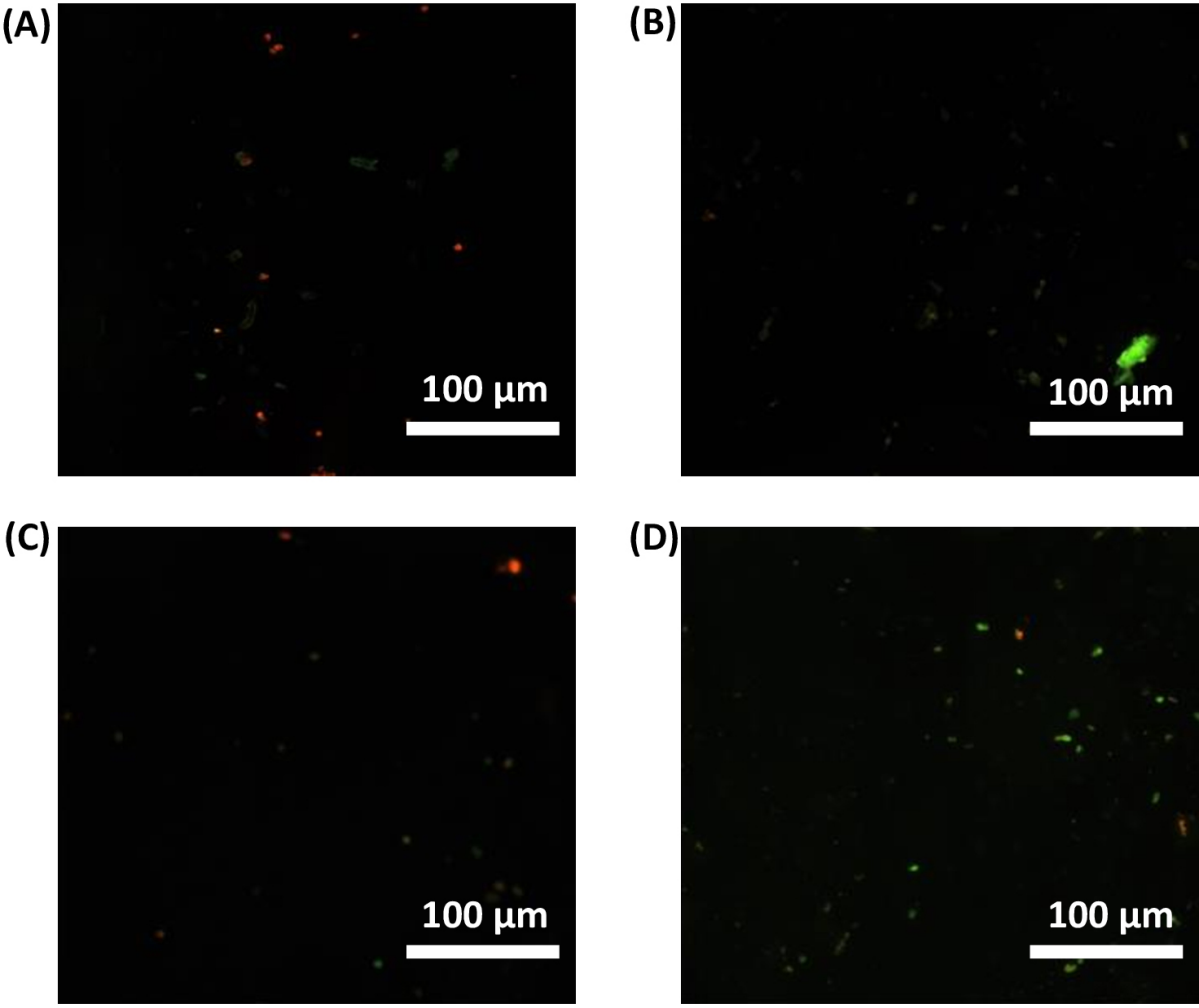
Live/ Dead assay (fluorescence microscopy). Live/Dead assay to check cytotoxicity of gelatin untreated (A), gelatin treated (B), gelatin-graphene oxide untreated (C), and microplasma treated gelatin-graphene oxide (D) using fluorescence staining methods (live/dead assay). Live cells and dead cells were fluorescently labelled green and red, respectively as visualized in the figure (scale bar 100 µm).

On comparing microplsma assisted hydrogel with traditional genipin crosslinked hydrogel for the cytotoxicity by MTT assay, microplasma treated gelatin hydrogel system showed significantly better cell viability even on 14th day of treatment as shown in Fig. 14.

#### Microscopy for cell-hydrogel interactions and cellular proliferation visualization

Untreated and microplasma-treated gel-GO hydrogels seeded with osteosarcoma cells of the MG63 osteosarcoma cell line were microscopically observed for up to 24 h. The effects of gelatin and GO on the MG63 cell line revealed important prospects by microscopy for this study. Microscopy showed (Fig. 15) better proliferation of the MG63 human osteosarcoma cell line in both gelatin- and gel-GO-treated hydrogel groups than those of the untreated gel groups.

#### Live/dead assay

We performed live/dead tests against MG-63 cells for all sample groups. We assessed the cytotoxicity of gelatin and gel-GO groups using a fluorescence staining method (live/dead assay). Live cells and dead cells were fluorescently labeled green and red, respectively. As shown in Fig. 16, almost all cells were found to be alive after a 24-h exposure to microplasma-treated gel-GO nanocomposite hydrogel.

**Figure 17:**
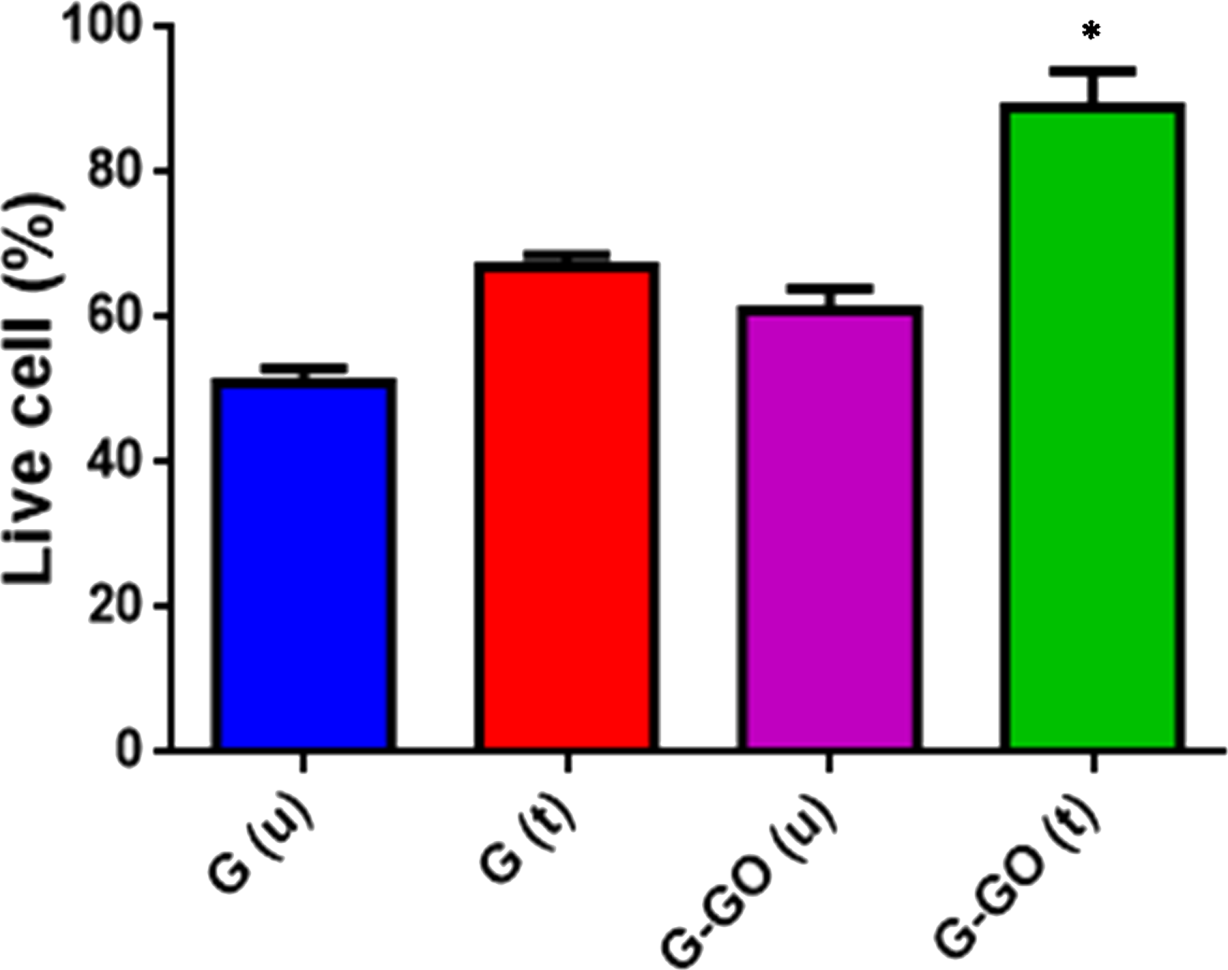
Live/Dead assay (graphical presentation). Quantitative Live/Dead assay to check primary biocompatibility and cell proliferation ability in presence of gelatin untreated G (u), gelatin treated G (t), gelatin-graphene oxide untreated G-GO (u), and microplasma treated gelatin-graphene oxide G-GO (t).

From quantitative live cell analysis under the fluorescence microscope at different fields, maximum cells (89%) were found to be alive with plasma treated gel-GO nanocomposite hydrogel as shown in Fig. 17.

## Discussion

**Figure 18:**
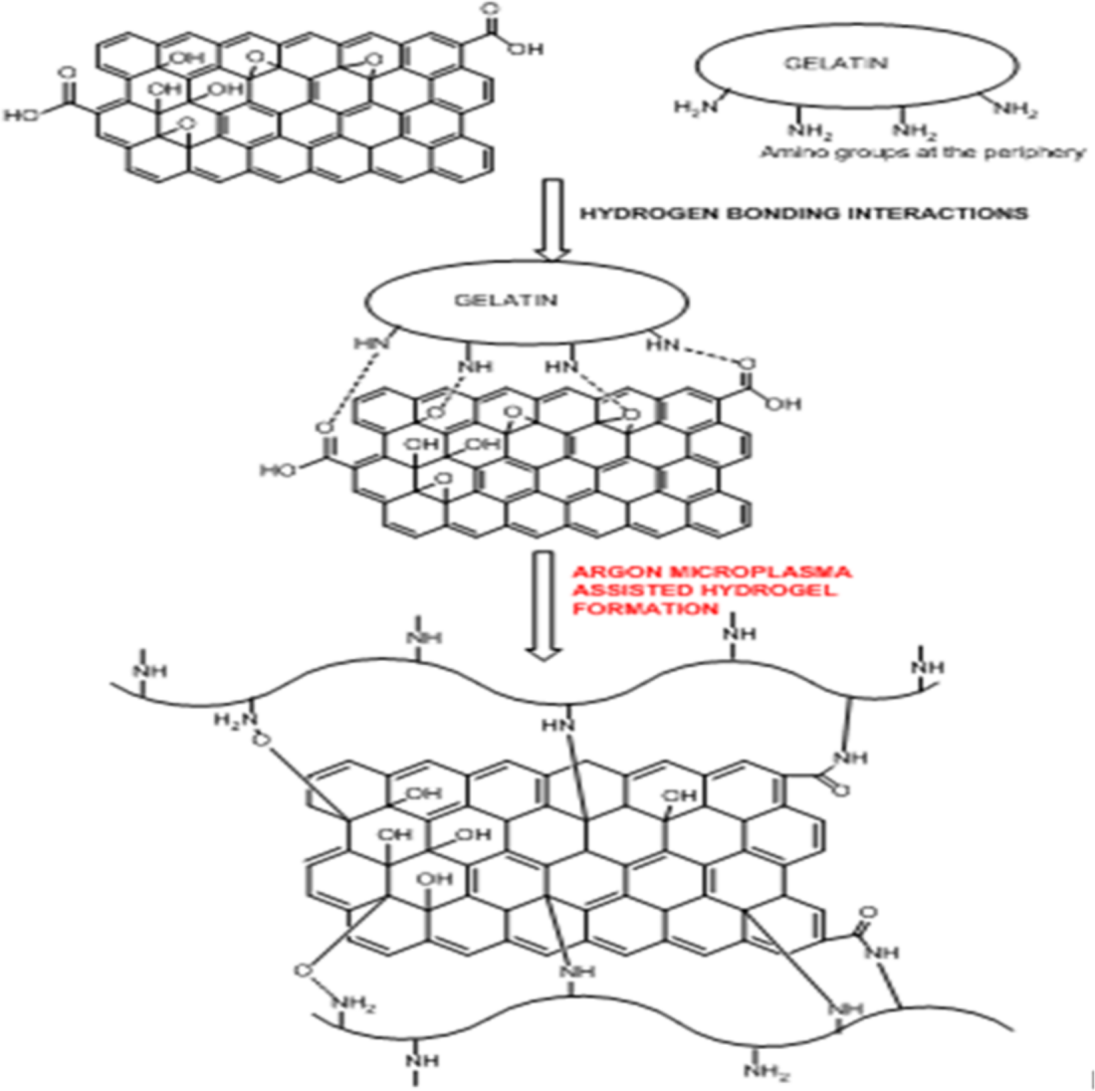
Molecular mechanism behind Ar-microplasma mediated gel-GO nanocomposite hydrogel synthesis. Schematic illustration of mechanism behind Ar-microplasma assisted gelatin-graphene oxide nano composite hydrogel synthesis.

Recently many polymeric hydrogel systems have been encouraged for biomedical applications and specifically in clinics (Censi et al., 2012; Shinde, Yeon & Jeong, 2013). The important characteristic properties to determine the quality of hydrogel systems such as mechanical strength, porosity, degradation kinetic, and bioactivity can be well tailored and controlled through chemical or physical methods (Duan et al., 2016; Kharkar, Kiick & Kloxin, 2013; Yesilyurt et al., 2016; Lim et al., 2014; Tibbitt et al., 2015). Apart from this, the components of the hydrogel as well as the environmental condition are prime important things interdependent on each other for successful fabrication of desired hydrogel material. Gelatin is the processed form of collagen to be used as suitable polymer due its peculiar characteristics such as it is a high molecular weight polypeptide and the primary protein component of animal connective tissues, such as bone, hide, skin and tendon (Nam & Park, 1999a; Nam & Park, 1999b). During hydrogel syntheses by chemical or physical processes, chemically crosslinked networks may result permanent junctions in irreversible manner, while physical networks have self modified controlled junctions that arise from either polymer chain entanglements or physical interactions such as ionic interactions, hydrogen bonds, or hydrophobic interactions (Jen, Wake & Mikos, 1996). To be more specific during physical methods of cross-linking, high energetic ionizing radiation such as gamma rays (Karadag et al., 2001) and electron beams (Ajji et al., 2008), has been profoundly used as hydrogels initiator providing threshold energy. The study was undertaken to assess the feasibility of microplasma as an emerging tool to fabricate crosslinked gel-GO nanocomposite hydrogels for biomedical applications. As illustrated in the Fig. 18 hydrogen bonding, interaction between gelatin and graphene oxide resulted the formation of gelatin-graphene oxide nano composite gel matrix. To make it stronger, elastic and viscous, further argon microplasma was used here in our study for free radical production and cross-linked elastic network formation by molecular entanglements and ionic hydrogen bonding or covalent interactions between gelatin polymer chains and graphene oxide molecules in gel-GO nano composite hydrogel synthesis.

The hydrogel system was characterized by cross-linking index measurements showing better cross-linking with increased porosity in the case of 8% purified gelatin. The large pore size and high porosity of the hydrogel materials enhanced the collision frequency of bio-macromolecules with free radicals produced from the plasma, which possibly helped promote the formation of cross-links between lysine and glutamic acid residues on gelatin chains along with the free and ionized radicals (Lai et al., 2013) produced by the highly energetic plasma.

In the case of 7% purified gelatin, pore size was smaller compared to that of 8% plasma-treated purified gelatin due to interactions of more water molecules present within it, with the excess free radicals produced during plasma treatment resulting in neutralization and a smaller extent of cross-linking. However, the overall mechanism and scientific artifacts are not yet clear. Also, compared to the dense structure of 10% purified gelatin, the 8% purified gelatin possessed increased contact areas between gelatin molecules leading to a higher cross-linking degree and larger pore size due to the ease of interactions. It was demonstrated that the scaffold pore size governs many things in orthopedic tissue engineering such as cellular encapsulation, attachment, organization, and delivery to maintain the cellular integrity to encourage natural reparative and regenerative processes of tissues. If the pore size is too small, pore blocking may occur by cells, and no further cellular penetration happens, thus inhibiting tissue growth and proliferation. In recent studies, the effective pore size for orthopedic tissue engineering purposes was reported to be in the range of 200∼350 µm for regeneration (Whang et al., 1999). SEM also confirmed better porosity to meet the orthopedic tissue engineering scaffold criteria to be extended for other tissue engineering applications. Confocal microscopy X–Z plane imaging confirmed GO encapsulation and gel-GO composite formation due to covalent cross-linking between amino groups of gelatin and carboxyl groups of GO at an optimized time of treatment, stirring speed, and temperature. Studies have shown that graphene and its chemical derivatives have the ability to support cellular proliferation, adhesion, and differentiation with little or no cytotoxic effects (Bai, Li & Shi, 2011; Shi et al., 2012).

Hydrogel swelling is an important parameter to determine the crosslinking density of hydrogels, and it affects cell adhesion and proliferation. Gelatin hydrogels should swell rapidly to a size sufficient to facilitate the attachment of cell grafts at implant sites. It was noted that the untreated gel-GO sample began to dissolve at 1 min due to weak viscoelastic and binding forces. Hydrogels used as tissue engineering scaffolds should not be quite dry, because the total water in the gel is comprised of both “bound” and “free” water (Hoffman, 2012). Our findings suggest that the cross-linked porous gel-GO prepared by plasma cross-linking seemed suitable for use as orthopedic tissue engineering scaffolds due to their stability and swelling capability in aqueous environments without structural disintegration for 1 h. Successful cross-linking of the gelatin and GO was confirmed by FTIR spectroscopy. FTIR measurements were conducted on un-cross-linked gelatin and GO and cross-linked gel-GO to determine whether cross-linking of the gelatin affected the primary gelatin structure. The FTIR spectrum of the un-cross-linked gelatin and GO revealed a structure that was quite similar to raw gelatin and GO after composite preparation. This demonstrated that Ar-microplasma did not greatly alter the structure of raw gelatin. This controlled microplasma method was found to provide good functionally additive energy during the cross-linking process, which is an essential condition to limit insolubility and increase the biocompatibility of gelatin for applications in the biomedical field over toxic chemical processes. Rheology confirmed that better viscoelasticity and stability were well maintained in the gel state at bodily physiological pH and temperature conditions. In both the untreated and plasma-treated groups, the good viscoelasticity and gel strength were possibly due to the addition of GO. The water contact angle measurements confirmed the decreased surface hydrophilicity of the plasma-treated gel-GO hydrogel films to make them more as a viscoelastic seal to attach to tissue surfaces for cellular adhesion and proliferation. This is attributed to Ar plasma treatment that induced modifications of the polymer to make it adequately hydrophilic. Water contact angle measurements proved it to be a suitable carrier material that provides a platform for cellular attachment and proliferation. The MTT assay revealed better proliferation with 1% GO in the composite hydrogel system even at up to 14 days compared to the untreated hydrogel groups. A higher number of covalent cross-links between adjacent polymer chains along with GO caused formation of a potential elastic network which is useful for cell penetration and growth. The additive binding forces for many-fold enhancement of the mechanical gel strength in treated samples were due to microplasma-induced cross-linking.

Cell proliferation positively indicated an initial adaptability of fibroblast cells, as well as cell attachment and adhesion. With an increase in the time period, cells began penetrating and proliferating due to the porous structure of the GO content in the composite hydrogel scaffolds. Furthermore, GO has several physiochemical properties such as an ultra-large surface area and possesses many functional groups including hydroxyl (OH), epoxy (C–O–C), and carboxyl (COOH) groups on its surface (Nezakati, Cousins & Seifalian, 2014). Therefore, if added to biomaterial tissue engineering scaffolds along with plasma as ionization energy source, GO can adsorb some biomolecules to improve their chemical and biological properties allowing bio-functionalization, and outstanding water solubility that makes the scaffold a promising material (Nguyen & Nguyen, 2016) for cellular proliferation. In our study we also compared our plasma treated gelatin scaffold system with traditionally synthesized genipin mediated crosslinked gelatin hydrogel system for cytotoxicity assay. Plasma mediated gelatin hydrogel was found to be with better cell viability and cellular proliferation for fibroblast cell line L929 and significantly with less cytotoxicity. The use of plasma process for tissue engineering polymer scaffold fabrication may be ideal for future biomedical application with less toxicity. Microscopy and the live/dead assay also observed with better results of cell proliferation and survival prospects with the hydrogel system.

It is noteworthy to mention for the first time that Ar-microplasma-induced gel-GO composite hydrogels supported cellular spreading and alignment with improved viability and proliferation. Suitable mechanical strength and enhanced tunable properties also represent desirable attributes of this composite hydrogel system, especially as a scaffold material in orthopedic tissue engineering. The resulting biodegradable, soft, elastic gel-GO nanocomposite hydrogel material was shown to cover a wide range of suitable properties for tissue engineering such as the cross-linking degree, pore size, hydrophilicity, viscoelasticity, and tunable mechanical properties; these are all imperative in controlling biological responses to implanted materials along with cells and growth factors at the defect site during healing and regeneration.

After characterizing the hydrogels, we obtained future insights for the application of our hydrogel system as a simple, novel, thin sealing plug material scaffold with the anticipation of better reliability during surgical interventions in orthopedic clinics for cartilage or bone regeneration as well as for biomedical tissue engineering without toxic chemical additives. The gel-GO hydrogel fabrication system with mechanical durability and improved cellular performance may provide an effective tool for the healing of complex defective and degenerative tissues.

## Conclusions

The present study optimizes the microplasma-mediated cross-linking process to overcome toxicity issues associated with fabrication of hydrogels in tissue engineering by chemical cross-linking . Further, this study explores the effect of Ar-microplasma in gelatin hydrogel formation containing GO. The gel-GO nanocomposite hydrogel was characterized by various methods such as the degree of cross-linking, FTIR spectroscopy, SEM, confocal microscopy, swelling behavior, contact angle measurement, and rheology, and the cell viability was also examined by an MTT assay, live/dead assay, and microscopy. The pore size of the hydrogel was found to be 287 ± 27 µm which is optimum for orthopedic tissue engineering purposes with future direction to be used in other tissue engineering fields. The contact angle of 78° ± 3.7°indicated the controlled hydrophilic nature of the hydrogel. Rheological data revealed improved storage as well as loss modulus of up to 50% with tunable viscoelasticity, gel strength, and mechanical properties at 37 °C body temperature conditions in the microplasma-treated groups. Better cell viability at 1% (w/w) of high functionality GO in gelatin was demonstrated by the MTT assay, microscopy, and live/dead assay as well as directly by inverted microscopy. As observed, the aforementioned plasma strategy is suitable to enhance the soft tissue engineering scaffold fabrication and tissue regeneration for promoting the clinical and biomedical applications in relevant fields. These encouraging results highlight the uniqueness of the Ar-microplasma process for gel-GO nanocomposite hydrogel scaffold fabrication and its promising attributes. This study moves forward the novel use of an electrically neutral beam of pure argon plasma from the bench top to the clinic for biomedical material fabrication with a tissue engineering approach to assist and accelerate the regeneration and repair of defective and damaged tissues. Keeping these exciting findings of the biomedical applicability endowed by Ar-microplasma in view, *in vitro* and *in vivo* experimental studies of hydrogels in various fields of basic and applied biomedical tissue engineering will be carried out in the near future. However, specific biomedical applications of plasma still require detailed investigations.

##  Supplemental Information

10.7717/peerj.3498/supp-1 Supplemental Information 1 Cross-linking Index Cross-linking index of various concentrations of purified gelatin. An asterisk indicates statistically significant difference of 8% purified gelatin group cross-linking (^∗^*P* > 0.05 and *n* = 5) as compared with other groups (7%, 9%, 10%).

10.7717/peerj.3498/supp-2 Supplemental Information 2 FTIR spectra analysis FTIR spectra of: original high functionality graphene oxide (red line), gelatin (blue line) untreated gelatin-graphene oxide gel sample (gray line) and Ar- microplasma treated gelatin-graphene oxide cross-linked sample (hydrogel) (yellow line). The FTIR spectrum of the un-cross-linked gelatin and graphene oxide revealed a structure that was quite similar to raw gelatin and graphene oxide after gelatine grapheme oxide nano-composite hydrogel preparation.

10.7717/peerj.3498/supp-3 Supplemental Information 3 Swelling property The swelling property of untreated Gel-GO matrix and microplasma treated Gel-GO nanocomposite hydrogels in deionized water (pH 7.4 and temp. 37 °C) showing significantly betterswelling property, stability and visco-elasticity of microplasma treated Gel-GO hydrogel in comparison to untreated Gel-Go material ( *n* = 3).

10.7717/peerj.3498/supp-4 Supplemental Information 4 Water contact angle (graphical) Water contact angle measurements of untreated Gel-GO nano composite material and microplasma treated Gel-GO hydrogel. ( *n* = 5).

10.7717/peerj.3498/supp-5 Supplemental Information 5 MTT assay (gel-GO untreated and gel-GO treated) Comparative MTT analysis of microplasma treated gelatin hydrogel and genipin cross-linked gelatin hydrogel by collection of liquid extraction medium at 1, 3 and 5 , 7,10 and 14 days of incubation with DMEM . MTT assay (L929 cell line) revealed the highest cell viability for microplasma treated gelatin hydrogel (^∗^*P* < 0.05; *n* = 6).

10.7717/peerj.3498/supp-6 Supplemental Information 6 MTT assay (plasma treated hydrogel and genipin treated hydrogel) Comparative MTT analysis of microplasma treated gelatin hydrogel and genipin cross-linked gelatin hydrogel by collection of liquid extraction medium at 1, 3 and 5 , 7,10 and 14 days of incubation with DMEM . MTT assay (L929 cell line) revealed the highest cell viability for microplasma treated gelatin hydrogel (^∗^*P* < 0.05; *n* = 6).

10.7717/peerj.3498/supp-7 Supplemental Information 7 Prisma checklist Summarised manuscript

10.7717/peerj.3498/supp-8 Supplemental Information 8 PRISMA flow diagram Schematic presentation of the gel-GO nano-composite hydrogel synthesis by Ar-microplsma and its biomedical application.

## Additional Information and Declarations

### Data Availability

The following information was supplied regarding data availability: The raw data has been supplied as Supplemental Files.
