# Supplementary material for: Microplasma-assisted hydrogel fabrication: A novel method for gelatin-graphene oxide nano composite hydrogel synthesis for biomedical application
Source: PeerJ. 2017 Jun 27;5:e3498. doi: 10.7717/peerj.3498 (PMC5490464; doi:10.7717/peerj.3498)
Supplement: Supplemental Information 7 — Summarised manuscript [file peerj-05-3498-s007.doc]

| **Section/topic** | **#** | **Checklist item** | | **Reported on page #** |
| --- | --- | --- | --- | --- |
| **TITLE: Microplasma-assisted hydrogel fabrication: A novel method for gelatin-graphene oxide nano composite hydrogel synthesis for biomedical application** | | | | 1 |
|  | 1 |  | Identify the report as a meta-analysis. |  |
| **Authors names and affiliations and address** | | | | 1 |
| **ABSTRACT** | | | |  |
| Structured summary | 2 | Words- 270 (Background, Rationale, Experimental, Findings, Applications and Prospects) | | 2 |
| **INTRODUCTION** | | | |  |
| Back ground and rationale | 3 |  | | 3-4 |
| Objectives | 4 |  | | 4-6 |
| **MATERIALS AND METHODS** | | | | 6-14 |
| Gelatin purification | 5 |  | | 6 |
| Preparation of GO (high functionality) | 6 |  | | 7 |
| GO characterization | 7 |  | | 8 |
| GO encapsulation in to gelatin matrix | 8 |  | | 8-9 |
| Synthesis of gel-GO hydrogel by Ar-microplasma | 9 |  | | 9 |
| Characterization of gel-GO hydrogel and primary *in vitro* study | 10 | Degree of cross-linking, Surface morphology by SEM, Spectral change observation by FTIR spectroscopy, Gel-GO nanocomposite visualization by confocal microscopy, Swelling behaviour, Rheological analyses, Water contact angle for hydrogel hydrophilicity | | 10-12 |
|  | 11 | 3-[4, 5-Dimethylthiazol-2-yl]-2, 5-diphenyl tetrazolium bromide (MTT) assay of cell proliferation | | 12-13 |
|  | 12 | Microscopy to observe cellular and hydrogel interactions | | 13-14 |
|  | 13 | Live/dead assay, | | 14 |
|  | 14 | Statistical analysis | | 14 |

| **Section/topic** | **#** | **Checklist item** | **Reported on page #** |
| --- | --- | --- | --- |
| **RESULTS** | | | 14-33 |
| GO characterization | 15 | GO characterization by XRD, Raman, XPS and TEM | 14-16 |
| Gel-GO hydrogel characterization | 16 | Cross-linking degree | 16-17 |
|  | 17 | Morphology and pore size analyses | 17-19 |
|  | 18 | FTIR analysis | 20-21 |
|  | 19 | Confocal microscopy | 22 |
|  | 20 | Swelling property | 23-24 |
|  | 21 | Rheology Microscopy | 24-26 |
|  | 22 | Water contact angle | 26-28 |
|  | 23 | MTT assay | 28-30 |
|  | 24 | Microscopy | 30-31 |
|  | 25 | Live/Dead assay | 31-33 |
| **DISCUSSION** | | | 33-39 |
| Novelty of the process and schematic illustration of the mechanism behind the process | 26 |  | 33-35 |
| Summary on the basis of evidences from results | 27 |  | 35-39 |
| Future insight | 28 |  | 39 |
| **CONCLUSIONS** | | | 40 |
| **REFERENCES** | | | 40-43 |
| Peer J End note style | 29 |  |  |
