# Supplementary material for: Microplasma-assisted hydrogel fabrication: A novel method for gelatin-graphene oxide nano composite hydrogel synthesis for biomedical application
Source: PeerJ. 2017 Jun 27;5:e3498. doi: 10.7717/peerj.3498 (PMC5490464; doi:10.7717/peerj.3498)
Supplement: Supplemental Information 8 — Schematic presentation of the gel-GO nano-composite hydrogel synthesis by Ar-microplsma and its biomedical application. [file peerj-05-3498-s008.docx]

**Prisma flow diagram**

## Validation

## Process selection

## Materials selection

Validation of the results and findings

Possible applications on the basis of valid results

Studies for material characterization

Studies for biomedical application of scaffold

## Application luded

Characterization

study

Process optimization

Fabrication of gel-GO nano-composite hydrogel system

Argon-microplasma as energy source

Gelatin and Graphene oxide

**Microplasma-assisted hydrogel fabrication: A novel method for gelatin-graphene oxide nano composite hydrogel synthesis for biomedical application**

Polymeric hydrogel scaffold for biomedical application

Safe and novel process selection for biomedical application in fabrication of scaffold

**
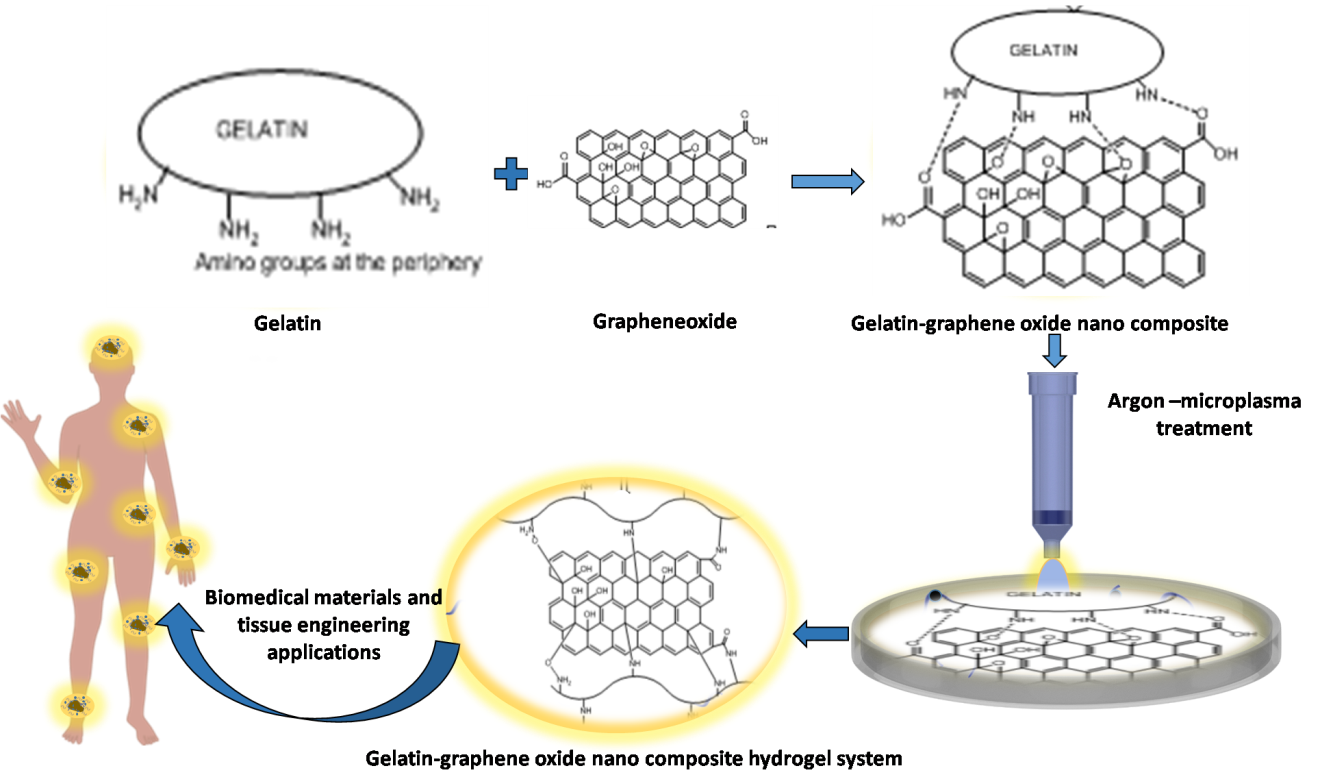
**
